# Papilloma-pseudovirus eradicates intestinal tumours and triples the lifespan of *Apc*^*Min/+*^ mice

**DOI:** 10.1038/ncomms15004

**Published:** 2017-04-11

**Authors:** Zhenyu Zhong, Yougang Zhai, Ping Bu, Shivanee Shah, Liang Qiao

**Affiliations:** 1 Department of Microbiology and Immunology, Stritch School of Medicine, Health Sciences Division, Loyola University Chicago, Maywood, Illinois 60153, USA; 2 Department of Ophthalmology, Stritch School of Medicine, Health Sciences Division, Loyola University Chicago, Maywood, Illinois 60153, USA; 3 Research Service, Edward Hines, Jr. VA Hospital, Hines, Illinois 60141, USA; 4 Biotherapy Center, The First Affiliated Hospital of Zhengzhou University, Zhengzhou, Henan 450052, China; 5 Institute of Precision Medicine, Jining Medical University, Jining, Shandong 272067, China

## Abstract

Inducing tumour-specific adaptive immunity, such as cytotoxic T lymphocyte (CTL) response, can result in promising antitumour effect against several human malignancies, especially in combination with immune checkpoint blockade strategies. However, little is known whether activation of innate immunity can lead to direct tumoricidal effect. Here, we develop a papilloma pseudovirus-based oral immunotherapeutic approach that shows strong tumoricidal effects in the gut, resulting in an almost tripled lifespan of *Apc*^*Min/+*^ mice (an animal model of human intestinal tumorigenesis). Mechanistically, these pseudoviruses activate the NLRP3 and AIM2 inflammasomes, leading to caspase-1-mediated tumour regression that is dependent on neither cytotoxic T lymphocytes nor humoral immune response. Blocking caspase-1 activation abrogated the therapeutic effects of the pseudoviruses. Thus, targeting innate immune sensors in tumours by the pseudoviruses might represent a strategy to treat intestinal tumours.

## Abstract (web summary)

Innate immunity sensors are expressed by both tumour cells and tumour-associated myeloid cells. Here, the authors show that stimulation of the innate immunity response with pseudoviruses in a genetic mouse model of intestinal cancer triggers tumour regression via Caspase-1 activation.

Being the key component of gut mucosa, intestinal epithelial cells (IECs) absorb nutrients, provide a niche for commensal bacteria and prevent invasion of harmful pathogens^1^. However, when the biogenesis of IECs is dysregulated, which often occurs as a result of the inherited genetic mutation, it can lead to the development of intestinal tumours. Conventional tumour vaccines aiming at inducing tumour antigen-specific immunity have shown limited efficacy in the clinical trials that is most likely due to the immunosuppressive microenvironment in the tumours.

Mucosal IECs and immune cells express a number of innate immune sensors, such as Toll-like receptors and Nod-like receptors (NLRs)^2^, for pathogen recognition. Activation of these immune sensors induces production of messengers (that is, chemokines/cytokines) that further recruit immune cells (for example, phagocytes, dendritic cells and adaptive immune cells) to join the battle against the invading pathogens^1^,^3^,^4^. In epithelial cell-derived tumours (for example, intestinal tumours), tumour cells and tumour-associated myeloid cells express Toll-like receptors/NLRs^4^,^5^. Expression of these receptors, in principle, offers ample opportunities to initiate an innate immune response, a critical prerequisite for inducing the adaptive immunities against tumours^2^. However, tumour-associated myeloid cells in the gut often produce immunosuppressive mediators that blunt the beneficial immune responses elicited upon vaccination^6^,^7^. It is therefore not clear whether intestinal tumour cells or tumour-associated myeloid cells can be targeted to induce mucosal immunity, and whether such immunity has a positive or negative role on intestinal tumour progression.

To address this question, we thought to induce both the innate and adaptive mucosal immunities in tumour-bearing animals and investigate whether and how such immunities may have an impact on tumour growth in the gut. We took advantage of the *Apc*^*Min/+*^ (multiple intestinal neoplasia) mice that have a mutation in the *Apc* gene and develop intestinal adenomatous polyps, a type of benign tumour that are mainly in the small intestine^8^. These mice, which serve as a well-characterized animal model for human familial adenomatous polyposis, can develop more than 60 benign polyps in their entire intestines by 5 months of age, and some of these polyps can eventually progress to adenocarcinomas^9^,^10^. We used pseudoviruses from papillomaviruses, which belong to a group of small DNA nonlytic viruses with skin- and mucosa-tropic properties^11^, as a tool to induce immune response against tumours in the *Apc*^*Min/+*^ mice. Papilloma pseudoviruses (PsVs) comprised a ‘shell' (also called virus-like particles (VLPs)) that is made of self-assembled papillomavirus viral L1 protein and a nonviral plasmid that is packaged inside the VLP^12^,^13^. The binding and uptake of PsVs or VLPs depends on heparan sulfate proteoglycans with a special conformation^14^. Consistent with this notion, PsVs and VLPs were recently shown to be capable of ‘pseudoinfecting' tumour cells but not healthy epithelial and mesothelial tissues, because nontumour cells lack the presence of such specifically modified heparan sulfate proteoglycans on their plasma membrane^15^. Due to their unique tropisms, we reason that pseudoviruses can serve as ideal ‘vehicles' for delivering protein antigens, which are either presented by VLPs or encoded by an encapsulated plasmid, to mucosal tumour and/or lymphoid tissues^16^,^17^,^18^,^19^. Although pseudoviruses do not self-replicate, they retain the immunogenicity of a virus due to the presence of capsid and DNA that are able to activate innate immunity^20^,^21^,^22^.

We hypothesize that, by infecting mucosal tumours and lymphoid tissues, PsVs would elicit antitumour innate and adaptive immunities to eradicate established tumours in the gut of *Apc*^*Min/+*^ mice. To test this hypothesis, we introduce a tumour-associated antigen (human carcinoembryonic antigen (hCEA)) into intestinal tumours of *Apc*^*Min/+*^ mice by crossing the *Apc*^*Min/+*^ mice with human CEA-transgenic (*hCEA-Tg*) mice^23^. The introduction of tumour-specific antigen would enable dissecting the roles of innate immunity versus tumour antigen-specific adaptive immunity in tumour regression. The progeny *hCEA-Tg/Apc*^*Min/+*^ mice have roughly similar kinetics of tumorigenesis as their parental *Apc*^*Min/+*^ mice^23^,^24^. Importantly, these mice have hCEA expression along the intestinal epithelia with specific elevation in intestinal tumour cells that mimics patients with familial adenomatous polyposis^24^,^25^. Our results show that PsV eradicates intestinal tumours of *Apc*^*Min/+*^ mice via activating the NLRP3 and AIM2 inflammasomes, leading to caspase-1-mediated tumour regression. Therefore, targeting innate immune sensors in tumours by PsVs might represent a strategy to treat intestinal tumours.

## Results

### PsV eradicates intestinal tumours in *Apc*^*Min/+*^ mice

**Figure 1:**
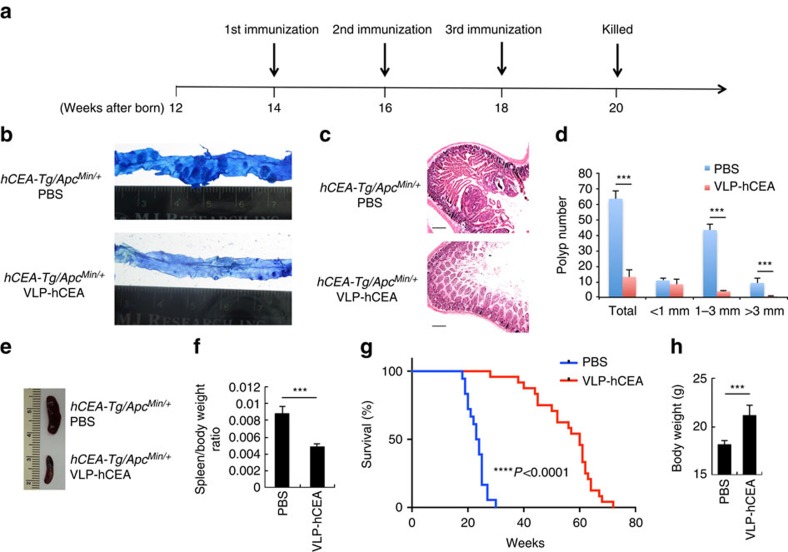
VLP-hCEA eradicates intestinal tumours and substantially extends the lifespan of *hCEA-Tg/Apc*^*Min/+*^ mice. (**a**) VLP-hCEA immunization scheme. (**b**) Methylene blue staining of the small intestines from *hCEA-Tg/Apc*^*Min/+*^ mice that were orally immunized with either PBS or VLP-hCEA pseudovirus. Ruler scale, 1 mm. (**c**) Representative haematoxylin and eosin (H&E) staining of small intestines after PBS or VLP-hCEA oral immunization in *hCEA-Tg/Apc*^*Min/+*^ mice. Scale bars, 200 μm. (**d**) The number of intestinal polyps of different sizes from *hCEA-Tg/Apc*^*Min/+*^ mice (*n*=7 for both groups, including 4 male and 3 female mice per group) that were immunized as in (**b**). Results are shown as mean±s.d. Student's *t*-test was performed to determine the statistical significance. Data are representative of three independent experiments. (**e**) Representative pictures of spleens from PBS or VLP-hCEA orally immunized *hCEA-Tg/Apc*^*Min/+*^ mice. (**f**) The ratios of mouse spleen to whole body weight of *hCEA-Tg/Apc*^*Min/+*^ mice (*n*=7 for both groups, including 4 male and 3 female mice per group) that were immunized as described in (**b**). Results are shown as mean±s.d. Student's *t*-test was performed to determine the statistical significance. Data are representative of three independent experiments. (**g**) Survival of *hCEA-Tg/Apc*^*Min/+*^ mice that were orally immunized with PBS (*n*=18, including 11 male and 7 female mice) or VLP-hCEA (*n*=24, including 13 male and 11 female mice). Log-rank test was performed to determine the statistical significance. Data are representative of two independent experiments. (**h**) Body weights of 20-week-old *hCEA-Tg/Apc*^*Min/+*^ mice that were orally immunized with either PBS (*n*=8, including 4 male and 4 female mice) or VLP-hCEA (*n*=10, including 6 male and 4 female mice). Results are shown as mean±s.d. Student's *t*-test was performed to determine the statistical significance. Data are representative of three independent experiments. ***P<0.001.

We made a PsV, which comprised bovine papilloma virus-like particles (made of viral L1 protein), with an hCEA-expressing DNA plasmid (pUMVC3-hCEA) packaged inside. This pseudovirus is hereafter referred to as VLP-hCEA. We first tested whether VLP-hCEA could induce tumour regression in *hCEA-Tg/Apc*^*Min/+*^ mice by orally gavaging *hCEA-Tg/Apc*^*Min/+*^ mice at 14 weeks of age when they had already developed numerous spontaneous polyps along the small intestine (Supplementary Fig. 1). After three rounds of immunization, with 2-week intervals between each immunization, we determined the antitumour efficacy of VLP-hCEA by counting the number of intestinal polyps 2 weeks after the final immunization (Fig. 1a). As shown in Fig. 1b–d, VLP-hCEA dramatically reduced the overall number of intestinal tumours in *hCEA-Tg/Apc*^*Min/+*^ mice. Notably, polyps that were >3 mm in diameter were completely eradicated after immunization (Fig. 1b,d). Moreover, the splenomegaly, one of the prognostic characteristics that positively correlate with intestinal tumour progression in *hCEA-Tg/Apc*^*Min/+*^ mice^23^, was reversed by VLP-hCEA immunization (Fig. 1e,f). Strikingly, only three doses of VLP-hCEA nearly tripled the lifespan of *hCEA-Tg/Apc*^*Min/+*^ mice (Fig. 1g). Consistently, VLP-hCEA treatment also prevented the weight loss in *hCEA-Tg/Apc*^*Min/+*^ mice (Fig. 1h).

### PsV eliminates tumours independent of adaptive immunity

To dissect the mechanism underlying the antitumour effect of VLP-hCEA, we first determined whether the adaptive immune responses, in particular the CD8^+^ T-cell response, played a role. VLP-hCEA immunization induced hCEA-specific T- and B-cell responses (Supplementary Fig. 2). Surprisingly, however, depleting CD8^+^ T cells before and throughout VLP-hCEA immunization did not affect the antitumour efficacy seen in *hCEA-Tg/Apc*^*Min/+*^ mice (Supplementary Fig. 3a,b). Moreover, the immune sera transferred from VLP-hCEA-immunized *hCEA-Tg/Apc*^*Min/+*^ mice also failed to reduce tumour numbers in unimmunized *hCEA-Tg/Apc*^*Min/+*^ mice (Supplementary Fig. 3c). These results suggest that VLP-hCEA-elicited cellular and humoral immune responses are dispensable for tumour regression.

**Figure 2:**
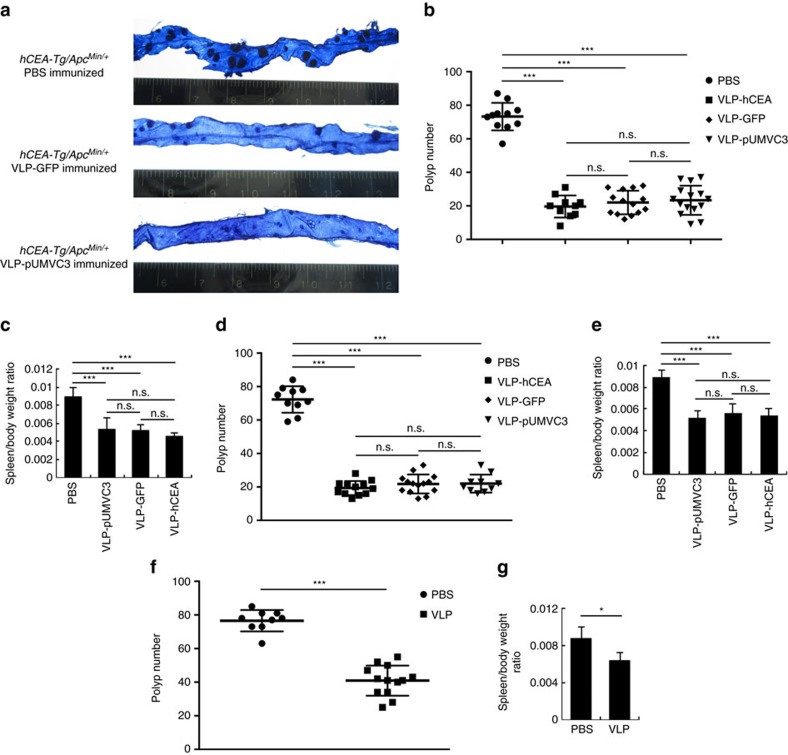
PsVs eradicate intestinal tumours in *hCEA-Tg/Apc*^*Min/+*^ mice independently of hCEA expression. (**a**) Methylene blue staining of the small intestines from *hCEA-Tg/Apc*^*Min/+*^ mice that were immunized with PBS, VLP-GFP or VLP-pUMVC3 pseudoviruses. (**b**) The overall numbers of intestinal polyps from *hCEA-Tg/Apc*^*Min/+*^ mice (*n*=10–15, including 6–8 male and 4–7 female mice per group) that were immunized as described in (**a**). Results are shown as mean±s.d. Data are representative of two independent experiments. (**c**) The ratios of mouse spleen to whole body weight of *hCEA-Tg/Apc*^*Min/+*^ mice that were immunized as described in (**a**). Results are shown as mean±s.d. Data are representative of two independent experiments. (**d**) Intestinal polyp numbers of *Apc*^*Min/+*^ mice (*n*=10–15, including 5–9 male and 5–6 female mice per group) that were immunized with PBS, VLP-hCEA, VLP-GFP or VLP-pUMVC3 pseudoviruses. Results are shown as mean±s.d. Data are representative of two independent experiments. (**e**) The ratios of mouse spleen to whole body weight of *Apc*^*Min/+*^ mice (*n*=7, including 3–4 male and female mice per group) that were immunized as described in (**d**). Results are shown as mean±s.d. Data are representative of two independent experiments. (**f**) Intestinal polyp numbers of *hCEA-Tg/Apc*^*Min/+*^ mice that were immunized with PBS (*n*=9, including 4 male and 5 female mice) or VLP (*n*=13, including 7 male and 6 female mice). Results are shown as mean±s.d. Data are representative of three independent experiments. (**g**) The ratios of mouse spleen to whole body weight of *Apc*^*Min/+*^ mice that were immunized as described in (**f**) (*n*=7, including 4 male and 3 female mice per group). Results are shown as mean±s.d. Data are representative of three independent experiments. Analysis of variance (ANOVA) was performed to determine the statistical significance in (**b**–**e**) and Student's *t* test was performed to determine the statistical significance in (**f**,**g**). *P<0.05; ***P<0.001; n.s., not significant.

Intriguingly, in comparison with those *hCEA-Tg/Apc*^*Min/+*^ mice that received VLP-hCEA immunization, we unexpectedly found a similar extent of tumour regression and splenomegaly reduction when they were treated with a control PsV that contain either a green fluorescent protein (GFP) plasmid (VLP-GFP) or an empty pUMVC3 plasmid vector (VLP-pUMVC3) (Fig. 2a–c). Notably, only VLP-hCEA induced anti-hCEA-specific immunity (Supplementary Fig. 2). These striking data indicate that neither the expression of hCEA nor generation of hCEA-specific immunity is needed for PsV-induced tumoricidal effect. To confirm this, we further tested whether removing hCEA in mice would also have no effect on the tumoricidal effect of VLP-hCEA. Indeed, oral administration of *Apc*^*Min/+*^ mice (no hCEA expression) with VLP-hCEA, VLP-GFP or VLP-pUMVC3 pseudoviruses eradicated the intestinal tumours and reduced splenomegaly to similar levels as found in the VLP-hCEA-immunized *hCEA-Tg/Apc*^*Min/+*^ mice (Fig. 2d,e). These completely unexpected results suggest that the hCEA-specific adaptive immunity in the host may not be essential for PsV-induced intestinal tumour regression. Interestingly enough, VLP alone (the empty viral shell without any DNA plasmid packaged inside) was also capable of reducing intestinal tumours and splenomegaly in *hCEA-Tg/Apc*^*Min/+*^ or *Apc*^*Min/+*^ mice, although the extent was only ∼40% of that induced by PsV (Fig. 2f,g). Together, these data indicate that hCEA-specific immunity is not necessary for PsV-induced tumour eradication. Instead, both the VLP and DNA plasmid packaged inside VLP appear to contribute to the tumoricidal effect in the gut.

### PsV activates the AIM2 and NLRP3 inflammasomes

**Figure 3:**
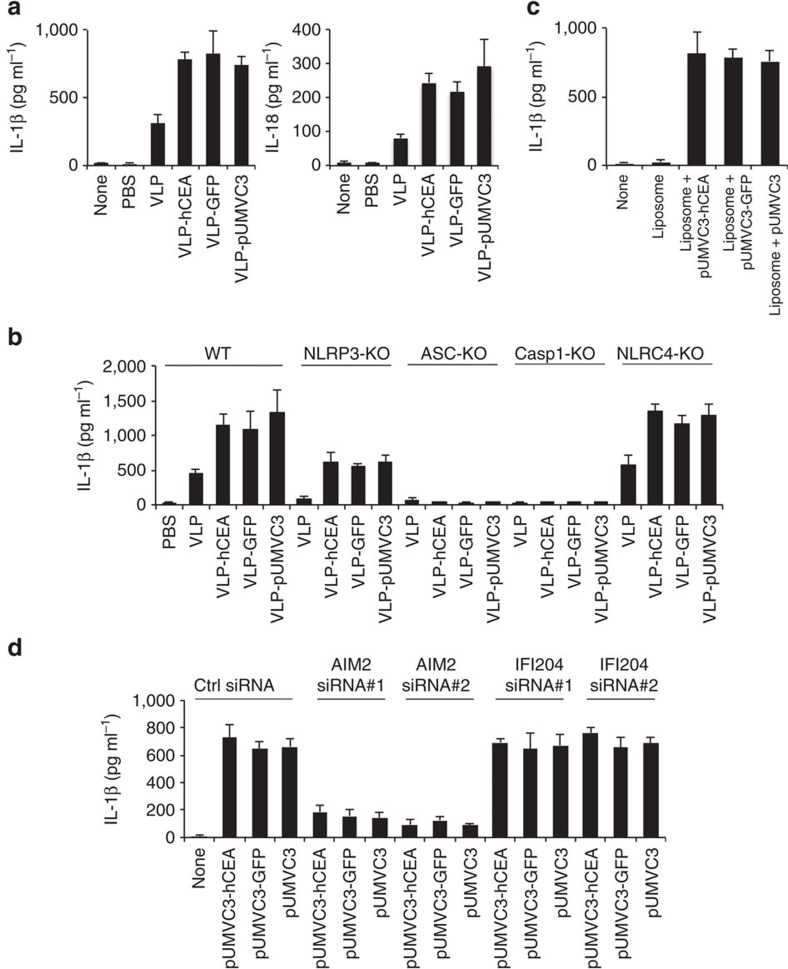
VLP and plasmid DNA activate NLRP3 and AIM2 inflammasomes respectively. (**a**) The levels of secreted IL-1β (left panel) or IL-18 (right panel) from small intestinal macrophages that were primed with LPS followed by stimulation with indicated stimuli. Results are shown as mean±s.d. (**b**) IL-1β levels from LPS-primed WT, NLRP3-KO, ASC-KO, Casp1-KO or NLRC4-KO immortalized BMDMs that were activated by the stimuli as indicated. Results are shown as mean±s.d. (**c**) IL-1β levels from LPS-primed BMDMs that were stimulated with indicated stimuli. Results are shown as mean±s.d. (**d**) IL-1β levels from LPS-primed WT or AIM2/IFI204 deficient BMDMs that were transfected with DNA plasmids as indicated. Results are shown as mean±s.d. All data are representative of three independent experiments.

We postulated that PsV might exert its tumoricidal effects via activating innate immunity. As VLP immunization was sufficient to induce antitumour effect (Fig. 2f,g), we decided to first study the innate immune responses induced by VLP. Previous studies reveal that particulate substances can trigger NLRP3 inflammasome activation, resulting in caspase-1 autoactivation and subsequent processing of proinflammatory cytokine pro-interleukin (pro-IL)-1β and pro-IL-18 (refs 26, 27, 28, 29, 30, 31). We therefore speculated that VLP, a particulate viral shell, might activate NLRP3 in a similar manner. To test this, we isolated macrophages from the small intestines of 14-week-old *Apc*^*Min/+*^ mice and then stimulated them with VLP, followed by measuring cytokine release 24 h post stimulation. We observed that VLP induced secretions of IL-1β and IL-18 from macrophages (Fig. 3a). *Apc* mutations can breach intestinal integrity, resulting in infiltration of bone marrow-derived mononuclear cells into the gut^32^. We therefore investigated whether VLP can also induce IL-1β and IL-18 release from bone marrow-derived macrophages (BMDMs). Like intestinal macrophages, we observed a similar response after VLP treatment (Supplementary Fig. 4a) in BMDMs. VLP-induced IL-1β and IL-18 release was largely dependent on NLRP3 inflammasome because macrophages deficient in NLRP3, ASC or caspase-1/11 abolished this effect (Fig. 3b). Mechanistically, we found that VLP required phagocytic uptake and lysosomal cathepsin B activity to activate inflammasome (Supplementary Fig. 4b,c). Moreover, VLP stimulation also induced mitochondrial reactive oxygen species (mtROS) production in myeloid cells (Supplementary Fig. 4d), and blockade of mtROS inhibited VLP-induced inflammasome activation (Supplementary Fig. 4e). Additionally, consistent with many known NLRP3 agonists^33^,^34^, the inhibition of ion fluxes also impaired VLP-induced inflammasome activation (Supplementary Fig. 4f). These data collectively indicate that VLP can engage NLRP3 inflammasome to activate caspase-1 and promote pro-IL-1β processing.

Interestingly, we found that PsV, similar to VLP, also triggered release of inflammasome-dependent cytokines from myeloid cells (Fig. 3a and Supplementary Fig. 4a), but with a higher extent. Because PsV and VLP have equal amounts of bovine papillomavirus L1 protein, we reasoned that the DNA plasmid packaged inside PsV may also contribute to IL-1β production after PsV stimulation. In support of this notion, deficiency in NLRP3 almost completely blocked VLP-induced IL-1β release, whereas only partially impaired PsV-induced IL-1β secretion (Fig. 3b). As ASC or caspase-1 deficiency completely abolished VLP- or PsV-induced IL-1β secretion (Fig. 3b), it is conceivable that the DNA plasmid packaged inside VLP was sensed via an NLRP3-independent but ASC/Caspase-1-dependent pathway. To further confirm that pUMVC3 plasmid alone can induce IL-1β secretion, we utilized liposomes to transfect macrophages with pUVMC3-hCEA, pUMVC3-GFP or pUMVC3 plasmids. As expected, these plasmids triggered caspase-1 activation and secretion of IL-1β, in the absence of VLP (Fig. 3c). To further identify the sensor that detects PsV DNA, we used small interference RNA (siRNA) to knock down AIM2 and IFI204 that were previously shown to sense cytosolic and nuclear DNA^35^,^36^,^37^, respectively. Although knocking down of IFI204 had minimal effect, ablating AIM2 expression almost completely abolished DNA plasmid-induced IL-1β release (Fig. 3d), suggesting that DNA plasmid inside PsV activates the AIM2 inflammasome. Taken together, our results indicate that VLP activates the NLRP3 inflammasome whereas PsV induces more potent inflammasome activation by engaging both NLRP3 and AIM2.

### Caspase-1 mediates PsV-induced tumour regression

**Figure 4:**
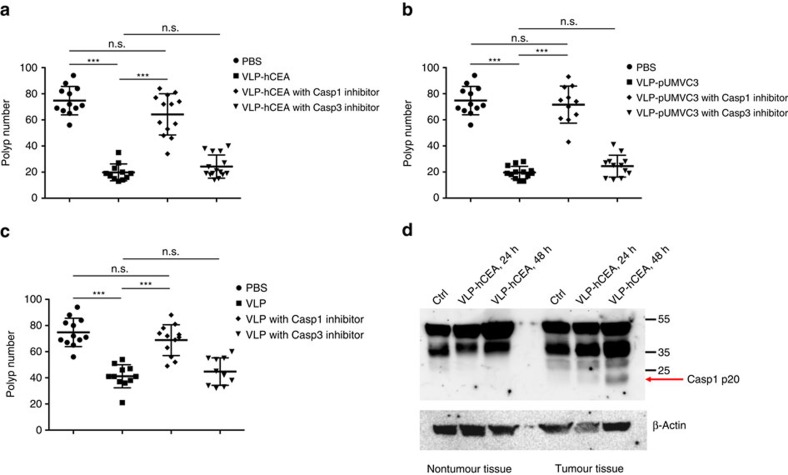
PsV or VLP-induced tumoricidal effect in the gut is mediated by caspase-1. (**a**–**c**) Intestinal polyp numbers of *hCEA-Tg/Apc*^*Min/+*^ mice (*n*=10–15, including 5–7 male and 5–8 female mice per group) that were pretreated with caspase-1 or -3 inhibitors followed by immunization of VLP-hCEA (**a**) VLP-pUMVC3 (**b**) or VLP (**c**). Results are shown as mean±s.d. Analysis of variance (ANOVA) was performed to determine the statistical significance. ***P<0.001; n.s., not significant. Data are representative of two independent experiments. (**d**) The 14-week-old *Apc*^*Min/+*^ mice were orally immunized with VLP-hCEA or PBS control. After 24 and 48 h, mice were killed and intestinal tissues were collected and separated microscopically as nontumour or tumour tissues. Cellular proteins were extracted and immunoblotted for caspase-1 or β-actin. Data are representative of three independent experiments.

As PsV induces potent inflammasome activation, which positively correlates with the remarkable antitumour efficacy, we reason that extent of PsV-induced caspase-1 activation and subsequent IL-1β/IL-18 release might be responsible for the antitumour effect induced by PsV immunization. As caspase-1 is a critical player for generating bioactive IL-1β and IL-18 (refs 38, 39, 40), we tested whether inhibiting caspase-1 may affect the tumoridical effect of PsV in the gut. Indeed, intraperitoneal (i.p.) injection of ZYVAD-FMK, a caspase-1 inhibitor, before PsV or VLP immunization abolished the tumoridical effect seen in *hCEA-Tg/Apc*^*Min/+*^ or *Apc*^*Min/+*^ mice. In contrast, inhibiting caspase-3 had little effect (Fig. 4a–c and Supplementary Fig. 5). Notably, caspase-1 inhibition did not affect the overall intestinal tumour load or size in unimmunized *hCEA-Tg/Apc*^*Min/+*^ or *Apc*^*Min/+*^ mice (Supplementary Fig. 6).

To further investigate the molecular mechanism underlying caspase-1-mediated tumoricidal effect, we first tested whether IL-1β and IL-18, two downstream cytokines of caspase-1 (refs 38, 39), contributed to PsV-induced tumour regression. IL-1β appears to be dispensable for eradicating intestinal tumours because genetic ablation of type-I IL-1 receptor in *hCEA-Tg/Apc*^*Min/+*^ mice did not compromise the antitumour efficacy of PsV (Supplementary Fig. 7a). IL-18 possesses antitumour effect, at least partially via regulating interferon-γ, signal transducer and activator of transcription 1 (STAT1) and IL-22 bind protein (IL-22BP)^41^,^42^. However, the blockade of IL-18 signalling by neutralizing antibodies, which effectively reduced gut IL-18 levels (Supplementary Fig. 7b,c), only had minimal influence on the tumoricidal effect of PsV in *hCEA-Tg/Apc*^*Min/+*^ mice (Supplementary Fig. 7d), consistent with a recent report showing a deleterious effect of IL-18 on gut barrier function^43^. Together, these results indicate that caspase-1-mediated non-IL-1/IL-18 pathway(s) is likely to drive PsV-induced tumour regression.

**Figure 5:**
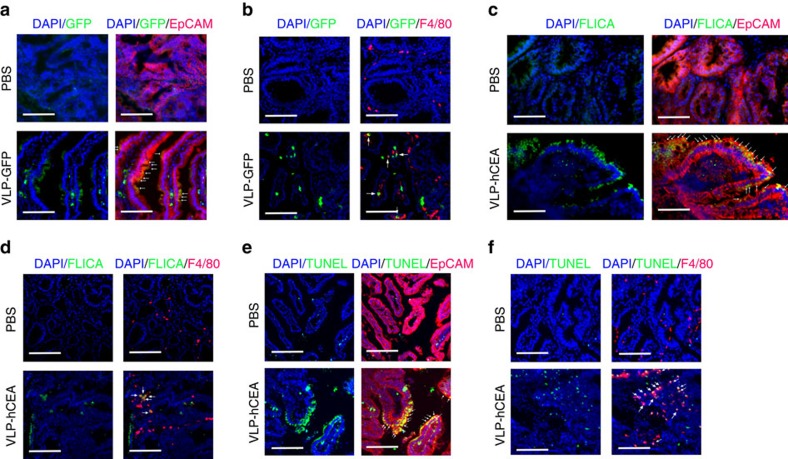
PsV induces pyroptosis in intestinal tumours. (**a**,**b**) The 14-week-old *Apc*^*Min/+*^ mice were orally immunized with VLP-GFP. At 48 h post immunization, the polyps of small intestines, which were separated microscopically from normal intestinal tissues, were collected and stained with anti-EpCAM, anti-GFP antibody and anti-F4/80 antibodies, respectively. Arrows indicate cells with colocalization of EpCAM/GFP or F4/80/GFP. Data are representative of three independent experiments. Scale bars, 100 μm. (**c**,**d**) The 14-week-old *Apc*^*Min/+*^ mice were orally immunized with VLP-hCEA. At 48 h post immunization, the polyps of small intestines, which were separated microscopically from normal intestinal tissues, were collected and stained with anti-EpCAM, anti-F4/80 antibodies and FAM-FLICA-Casp1 probe, respectively. Arrows indicate cells with colocalization of EpCAM/FLICA or F4/80/FLICA. Data are representative of three independent experiments. Scale bars, 100 μm. (**e**,**f**) The 14-week-old *Apc*^*Min/+*^ mice were orally immunized with VLP-hCEA. At 48 h post immunization, the polyps of small intestines, which were separated microscopically from normal intestinal tissues, were collected and co-stained with anti-EpCAM (**e**) or anti-F4/80 (**f**) antibodies and TUNEL, respectively. Arrows indicate dying intestinal tumour cells or macrophages. Data are representative of three independent experiments. Scale bars, 100 μm.

Caspase-1 is known to mediate an inflammatory form of cell death, named pyroptosis^44^,^45^. We next investigated whether PsV could directly induce intestinal tumour cell death via activating caspase-1. To this end, we orally gavaged 14-week-old *Apc*^*Min/+*^ mice with a single dose of VLP-GFP and analysed the intestinal tumour and nontumour tissues 24 and 48 h post immunization. As shown in Fig. 4d (also Supplementary Fig. 11), PsV induced pro-caspase-1 processing (p20 fragment) only in tumour tissues that was more pronounced 48 h after immunization. In line with this notion, we performed immunofluorescent staining and confirmed that PsV indeed infected intestinal tumour cells in *Apc*^*Min/+*^ mice *in vivo* (Fig. 5a). As expected, PsV was also taken up by intestinal macrophages (Fig. 5b). Importantly, PsV-infected intestinal tumour cells and macrophages had active caspase-1 48 h after oral gavage immunization, suggesting a rapid ongoing pyroptosis *in vivo* that was not observed in phosphate-buffered saline (PBS)-treated group (Figs 4d and 5c,d). Consistently, we observed significantly increased number of dying intestinal tumour cells and macrophages by TUNEL (TdT-mediated dUTP nick end labelling) staining 48 h after PsV immunization (Fig. 5e,f).

Induction of intestinal tumour cell death by PsV immunization raised the concern whether PsV might compromise intestinal barrier function, leading to increased translocation of gut microbes that promote inflammation and development of inflammatory bowel disease. We therefore determined whether the gut permeability was compromised after PsV or VLP immunization. To this end, we quantified the levels of faecal albumin, an indicator for gut permeability, before and after PsV or VLP oral gavage immunization. As shown in Supplementary Fig. 8, no significant changes in faecal albumin levels were found between VLP-hCEA- and PBS-treated mice during the first two immunizations, indicating that the intestinal barrier function is not affected by vaccination. Interestingly, VLP-hCEA treatment eventually even led to a better preservation of gut integrity relative to PBS-treated mice (Supplementary Fig. 8), which positively correlates with the reduced tumour formation and enhanced survival of after immunization.

The above results indicate that PsV- or VLP-induced caspase-1 activation mediates intestinal tumour regression, and we therefore reason that increasing immunization frequency might yield an even better tumoricidal efficacy. Indeed, 6 doses immunizations (8-week apart between the two consecutive 3-dose immunizations) with VLP-pUMVC3 significantly extended the lifespan of *Apc*^*Min/+*^ mice compared with those receiving only 3 doses of VLP-pUMVC3 immunization (Supplementary Fig. 9a). The improved lifespan of *Apc*^*Min/+*^ mice positively correlates with the enhanced initial tumour eradication efficacy compared with the 3-dose immunization (Supplementary Fig. 9b).

### PsV-induced CTL prevents tumour relapse

**Figure 6:**
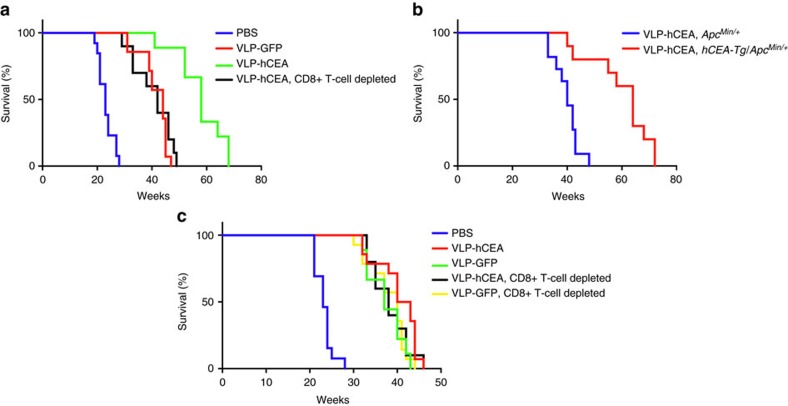
PsV-induced tumour antigen-specific CTL response prevents tumour relapse. (**a**) Survival of *hCEA-Tg/Apc*^*Min/+*^mice that were immunized (one round of three-dose immunization) with VLP-GFP or VLP-hCEA. One group of mice were pretreated with anti-CD8a antibody to deplete CD8^+^ T cells before VLP-hCEA immunization (*n*=9–14, including 5–7 male and 4–7 female mice per group). Data are representative of two independent experiments. (**b**) Percentage of survival of *Apc*^*Min/+*^ (*n*=11, including 7 male and 4 female mice) or *hCEA-Tg/Apc*^*Min/+*^ (*n*=10, including 6 male and 4 female mice) mice that were immunized (one round of three-dose immunization) with VLP-hCEA. Data are representative of three independent experiments. (**c**) The percentage of survival of *Apc*^*Min/+*^ mice that were immunized (one round of three-dose immunization) with VLP-hCEA or VLP-GFP. Two groups of mice were pretreated with anti-CD8a antibody before VLP-hCEA or VLP-GFP immunization (*n*=9–14, including 5–8 male and 4–7 female mice per group). Data are representative of two independent experiments. Log-rank test was performed to determine the statistical significance in (**a**–**c**).

Interestingly, although dispensable for the initial tumour eradication, hCEA-specific immunity delayed tumour relapse after the initial tumour regression in *hCEA-Tg/Apc*^*Min/+*^ mice. This was evidenced, at least partially, by the survival advantage of *hCEA-Tg/Apc*^*Min/+*^ mice vaccinated with VLP-hCEA as compared with those immunized with VLP-GFP (Fig. 6a). Furthermore, the hCEA-specific protection against intestinal tumour relapse was likely mediated by CTLs because depleting CD8^+^ T cells before and throughout VLP-hCEA immunization approximately reduced the lifespan of *hCEA-Tg/Apc*^*Min/+*^ mice to that of VLP-GFP-immunized mice (Fig. 6a). Consistent with the finding that hCEA-specific CD8^+^ T-cell response prevented intestinal tumour relapse, we found that hCEA expression by intestinal tumour cells was a crucial determinant for preventing tumour relapse because VLP-hCEA immunization led to the enhanced survival of *hCEA-Tg/Apc*^*Min/+*^ mice (with hCEA expression in intestinal polyps) relative to that of *Apc*^*Min/+*^ mice (no hCEA expression) (Fig. 6b). To further support this concept, VLP-hCEA- or VLP-GFP-immunized *Apc*^*Min/+*^ (lacking hCEA expression) had similar survival time with or without depletion of CD8^+^ T cells (Fig. 6c). Together, these results indicate that, although dispensable initial tumour eradication, PsV-induced tumour antigen-specific CTL response prevents tumour relapse and extends lifespan of the animal.

## Discussion

We have demonstrated that only three doses of PsV immunization can induce remarkable tumour regression and almost triple the animal survival time. Intriguingly, although PsV (that is, VLP-hCEA) indeed induced humoral and cellular immune responses against the tumour antigen, hCEA, neither CD8^+^ T cells nor antibodies are required for the PsV-induced tumour regression. Instead, tumour elimination and prolonged animal survival are mainly achieved via activating the innate ‘inflammasome-caspase-1' pathway. Intriguingly, although dispensable for PsV-induced initial tumour eradication, tumour antigen (hCEA)-specific CD8^+^ T-cell response plays a role in preventing tumour relapse, thereby further prolonging the lifespan of the tumour-bearing mice. Our study expands our current knowledge of cancer immunotherapy that, in addition to adaptive immunity, activation of innate immune pathways can also yield effective antitumour activity, thereby providing a basis for future development of a similar approach to combat cancer in patients. Many types of tumour cells and tumour-associated myeloid cells express inflammasome sensors^4^,^5^, and it is likely that engaging these innate immune signalling pathways might be beneficial for the treatment of other cancers.

Our results suggest that PsV-induced innate immunity exerts antitumour effect before the induction of adaptive immunity (activation of inflammasome and death of tumour cells can be seen within 48 h after PsV immumization). Moreover, PsV is known to infect macrophages and dendritic cells^22^. We speculate that due to the change in intestinal tumour microenvironment after PsV infection, tumour-specific mucosal CTLs induced by PsV immunization might be able to work in a more favourable milieu, thereby preventing tumour relapse and extend the lifespan of tumour-bearing mice. In support of this notion, we found that a single dose of VLP-hCEA treatment resulted in downregulated expression of a number of immune genes in the tumour tissues that favoured tumour growth (that is, IL-6, transforming growth factor-β, IL-10, IL-23A, programmed death-ligand 1, tumour necrosis factor-α, IL-17A and Foxp3)^46^,^47^,^48^,^49^, although not affecting the ones that suppress tumour growth (that is, IL-12, interferon-γ, IL-18 and iFi204)^47^,^48^,^49^,^50^ (Supplementary Fig. 10). We also found that the expression of M2/tumour-associated macrophage (TAM)-associated genes (Arginase and Ym-1)^47^,^48^ was reduced after VLP-hCEA treatment, while the one (that is, iNOS) that associates with M1 macrophage phenotype^47^,^48^ was not affected (Supplementary Fig. 10). Further study is needed to investigate the possible causal relationship between change in tumour microenvironment and CTL-mediated anti-tumour immunity.

Although normal epithelial cells express both NLRP3 and AIM2 innate immune receptors, we observed significant pyroptosis primarily in intestinal tumour cells but not normal epithelial cells. We speculate that this might be due to a combination of the following reasons. First, it was recently shown that PsV and VLP can preferentially infect tumour cells^15^ that could explain the much enhanced pyroptosis in tumour cells versus normal intestinal epithelial cells. Moreover, we have demonstrated that PsV activates the inflammasomes, at least partially, via ROS. It was shown that intestinal tumour cells have high levels of ROS^51^,^52^, and we therefore postulate that PsV further enhances production of ROS in the tumour cells, making them more sensitive to inflammasome activation and consequent pyroptosis. Additionally, it is also likely that drastic reduction of mucus deposition surrounding intestinal tumours might make them more accessible to PsV or VLP infection^32^,^53^,^54^, thereby increasing the load of PsV or VLP that subsequently enhances the magnitude of the PsV- or VLP-induced inflammasome activation and pyroptosis. It should be noted that, although PsV induces a remarkable antitumour effect, our results cannot distinguish whether PsV vaccination prevents tumour initiation or slows down progression, or both, in *Apc*^*Min/+*^ mice. In addition, we cannot completely exclude the possibility that PsV-induced caspase-1 activation might somehow shape the composition of gut microbe community that might contribute to tumour regression. Further studies are needed to answer these questions.

Preexisting immunity is a major concern when a viral vector is used to treat patients as the preexisting antibodies might neutralize the vector, thereby limiting its efficacy^55^,^56^. Although VLP can induce vector-specific IgA in the intestinal washings^57^, it clearly did not reduce the efficacy of PsV because two rounds of a three-dose consecutive immunization induced a more remarkable tumour regression and better survival than only one round. This indicates that PsVs are not effectively neutralized by IgA and thus may be used repeatedly, a great advantage for long-term therapy. As the tumours are at the apical surface of the intestines, it is possible that the PsV coated with the IgA may still be able to infect tumours, in particular the macrophage population that are associated with tumour cells.

Lastly, immunotherapies with antibodies against CTLA4, PD1 and PDL1/2 have resulted in significant benefit to cancer patients^58^,^59^,^60^,^61^, a rising hope for patients with advanced tumours. By blocking these negative checkpoints, host adaptive immunity may function at the desired efficacy to control tumour growth. However, not all tumours are immunogenic enough to elicit tumour antigen-specific immune responses even when the negative check points are blocked^62^. This may explain ineffectiveness of these antibodies in certain patients, including the ones with colorectal cancer. As our PsV therapeutic strategy targets innate immunity as well as adaptive immunity against tumours, combination of PsV with CTLA-4/PD-1/PD-L1/2 blockade approaches is expected to yield promising success in patients who failed in these checkpoint blockade therapies.

## Methods

### Mice

*C57Bl/6* mice, *Ilr1*^*−/−*^ mice and *Apc*^*Min/+*^ mice were purchased from The Jackson Laboratory (Bar Harbor, ME, USA). *hCEA-Tg* (CEA.Tg, Line 2682, *C57Bl/6* (H-2b), heterozygous) mice were originally generated from Dr John Thompson (University of Freiburg, Freiburg, Germany) and kindly provided by Drs John W. Greiner and Jeffrey Schlom (National Cancer Institute, NIH, Bethesda, MD, USA). Transgenic mice were generated from *C57Bl16* X *CB6* F1 mice (Ciba Animal Breeding Center, Basel, Switzerland) and lines were established from founder animals by mating with *C57Bl16* mice^25^. The *hCEA-Tg* and *Apc*^*Min/+*^ mouse colonies were maintained by continuous backcrossing with *C57Bl/6* mice since 1999. *hCEA-Tg/Apc*^*Min/+*^ mice were generated by crossing male *Apc*^*Min/+*^ mice with female *hCEA-Tg* mice^23^. *hCEA-Tg/Apc*^*Min/+*^*/Il-1r1*^*−/−*^ mice were generated by crossing the *hCEA-Tg/Apc*^*Min/+*^ mice with *Ilr1*^*−/−*^ mice. All mice were maintained under specific pathogen-free conditions. All experimental procedures were carried out according to the protocols approved by the institutional animal care and use committee.

### Reagents

ATP, phorbol myristate acetate, cytochalasin D and BAPTA-AM were from Sigma-Aldrich. Ultrapure lipopolysaccharide (LPS) was from Invivogen. DPI was from Calbiochem. Lipofectamine 2000 and MitoSOX were from Life Technologies. Calcium-free and calcium-containing Dulbecco's modified Eagle's medium (DMEM) were from US Biologicals. Caspase-1 inhibitor Z-YVAD-FMK and caspase-3 inhibitor Z-DEVD-FMK were from Enzo Life Sciences. The lactate dehydrogenase assay kit and *in situ* cell death detection kit (TUNEL) were from Roche. FLICA capase-1 kit was from Immunochemistry. Antibodies used for immunoblotting were as follows: anti-mouse caspase-1 (AG-20B-0042-C100, Adipogen, 1 μg ml^−1^), anti-mouse β-actin (sc-1615 horseradish peroxidase (HRP), Santa Cruz Biotechnology, 0.1 μg ml^−1^) and anti-mouse IL-1β (AF-401-NA, R&D Systems, 0.25 μg ml^−1^). Imject Alum and streptavidin-HRP were from Pierce.

### Generation of papillomavirus virus-like particles and pseudoviruses

The C-terminal truncated bovine papillomavirus-1 (BPV-1) L1 capsid protein were used to generate VLP using the recombinant baculovirus expression system and purified as previously described^17^. The hCEA expression plasmid pUMVC3-hCEA encoding human carcinoembryonic antigen without the NH2-terminal signal peptide was constructed by ligation of *Eco*RI and *Not*I enzyme digested hCEA gene fragment from pCI-CEA plasmid^16^ with pUMVC3 vector plasmid. The CEA gene sequence and protein expression were verified by sequencing and immunoblot analysis. BPV L1 pseudoviruses were generated as previously described^16^. Briefly, purified VLP were dialysed against 10 mmol l^−1^ HEPES solution, and then VLPs (40 μg) were disrupted at the condition of 25 mmol l^−1^ Tris-HCl (pH 8.0), 15 mmol l^−1^ NaCl, 10 mmol l^−1^ EGTA and 20 mmol l^−1^ dithiothreitol in a final volume of 200 μl at room temperature for 60 min. Then, 1 μg μl^−1^ plasmid pUMVC3-hCEA, pUMVC3-GFP (expression GFP) or pUMVC3 vector (20 μl) was added and the mixture was diluted by 220 μl reassembly buffer containing 25 mmol l^−1^ CaCl_2_ and 20% dimethyl sulfoxide to form pseudoviruses at room temperature for 4 h. The reassembled pseudovirus particles were verified by Zeiss EM900 electron microscopy as described previously^17^. The efficiency of plasmid DNA encapsidation in the VLP was analysed by measuring the amount of DNA plasmid inside of the VLP as described previously^16^. Briefly, VLP-hCEA or VLP-pUMVC3 pseudovirus preparation (100 μl) was treated with 80 units Benzonase (Sigma) for 1 h at 37 °C and heated at 100 °C for 10 min, and then digested with proteinase K (1 mg ml^−1^) at 55 °C for 3 h. The remaining plasmid DNA was extracted and the amount of plasmid DNA was determined by ultraviolet spectrophotometry quantitation. The amount of the plasmid DNA packaged inside `VLP was used to determine the copy numbers of the PsV. To ensure the quality of purified VLP, endotoxin level in the batches of purified VLPs was analysed using Limulus Amebocyte Lysate Chromogenic Endotoxin Quantitation Kit (Pierce). VLP batches with endotoxin level <1 EU ml^−1^ were directly used for PsV preparation. Contamination of sf9 host cell proteins in the batches of purified VLPs was analysed by enzyme-linked immunosorbent assay (ELISA) using anti-sf9 host cell protein antibody (Abcam). Contamination of host cell proteins was measured as 5–10%. Host DNA contamination in disrupted VLP solution was analysed by agarose gel electrophoresis and SYBR green staining (Invitrogen), and no detectable host DNA was found.

### Cell culture and stimulation

BMDMs were generated by culturing the mouse bone marrow cells using DMEM complete medium in the presence of 20% vol/vol L929 conditional medium^26^,^63^. Immortalized murine macrophages *from Nlrp3*^*−/−*^, *Asc*^*−/−*^, *Capase-1*^*−/−*^, *Nlrc4*^*−/−*^, *Cathepsin B*^−/−^ mice and their corresponding wild-type control cells were generously provided by Dr Katherine Fitzgerald and as previously described^26^, and were routinely tested for mycoplasma contamination by using Mycoplasma Detection Kit (ThermoFisher Scientific). After pretreatment with ultrapure LPS (100 ng ml^−1^) for primary or immortalized BMDM, the cells were then stimulated with ATP for 30 min and VLP or pseudovirus for 18 h. Plasmids were transfected into macrophages using Lipofectamine 2000 according to the manufacturer's instructions. In the experiments using chemical inhibitors, they were added 1 h before inflammasome agonists. After inflammasome agonist stimulation, culture supernatants and cell lysates were collected for ELISA and immunoblot analysis.

### Vaccination and sample collection

The 14-week old *Apc*^*Min/+*^, *hCEA-Tg/Apc*^*Min/+*^ or *hCEA-Tg/Apc*^*Min/+*^*/Il-1r1*^*−/−*^ mice were used for the therapeutic vaccination experiments. No randomization or blinding was performed. For oral immunization, mice were immunized three times at a 2-week interval by oral gavage with either PBS, 20 μg BPV VLP or 0.8–1 × 10^11^ VLP-hCEA, VLP-pUMVC3 or VLP-GFP pseudovirions generated from 20 μg BPV VLP. In another set of immunization, mice were orally immunized three times as described above and received another round of three-time immunization 8 weeks after the first round of immunization. At 2 weeks after the third immunization or after the second round of three-time immunization, mice were killed and serum, intestinal washing and whole small intestines were collected from individual mouse for the subsequent experiments. Faecal samples were collected individually before mice killing. For caspase inhibitor treatment, mice received caspase-1 inhibitor Z-YVAD-FMK (5 mg/kg of body weight in 150 μl volume), caspase-3 inhibitor Z-DEVD-FMK or the corresponding diluent (3% dimethyl sulfoxide in PBS) by i.p. injection on days −1, 0 and 1 of each immunization and every 3 days during the interval of immunization. The mice were under treatment till 2 weeks after final immunization.

### Spleen to body weight ratio and survival

After killing, mice body weight was measured, and then mice spleens were collected and spleen weight was measured. The ratios of spleen to body weight were calculated as spleen weight/body weight. Mice body weights were measured 2 weeks after the third immunization (20-weeks old). Mice survival (whether found dead or killed) was monitored every other day after immunization. When they became moribund, they were considered to have reached the experimental end point and were then killed.

### Isolation of lymphocytes from spleen and Peyer's patches

Lymphocytes from spleen and Peyer's patches were isolated as previously described^16^. Briefly, mouse spleen was homogenized and followed by treatment with ACK lysis buffer to lyses red blood cells. The single-cell suspension was collected by passing splenocytes through 70 μm cell strainers (BD Biosciences, Bedford, MA, USA). Then, the cell suspension was incubated in nylon-wool columns (Polysciences, Warrington, PA, USA) at 37 °C for 1 h and the enriched T cells were washed through the columns with complete RPMI-1640. To isolate lymphocytes from Peyer's patches, visible Peyer's patches were collected from ice-cold PBS-flushed small intestines. After homogenization, cells were passed through 70 μm cell strainers and lymphocytes were isolated by Ficoll gradient separation (GE Healthcare).

### Tumour quantification and histological analysis

Methylene blue dye was used for staining of intestinal tumours. Briefly, mouse small intestines were collected from each group and flushed with cold PBS to remove the faecal contents. Intestines were then opened and rinsed in 1% methylene blue dye for 1 h at room temperature. The excessive methylene blue dye was washed out by rinsing the intestines with PBS overnight. The tumour numbers and sizes were assessed via measuring the deep stains in intestine by an investigator blinded to the treatment groups. The staining results were recorded with photographs. The small intestine was divided into three equal parts and tumour numbers were counted and grouped based on sizes: <1 mm, 1–3 mm and >3 mm. Intestinal tissues with tumours were used for histological analysis. Tissue sections of 5 μm thick were stained with haematoxylin and eosin and assessed by a pathologist blinded to the experimental groups. Presence of tissue disorganization, dysplasia, mitosis, invasion of adjacent normal tissues, inflammatory cell infiltrates and tissue necrosis was examined and compared among the groups.

### *In vivo* depletion of CD8^+^ T cells and serum transfer

For CD8^+^ T-cell depletion, mice were i.p. injected with 250 μg of anti-CD8a IgG2a antibody (from Cedarlane) at 2 days and 1 day before every pseudovirus immunization. During the interval of immunization, mice were given additional 250 μg of anti-CD8a antibody every 3 days until the end of the experiment. Control mice were i.p. injected with 250 μg of IgG2a isotype control antibody with the same schedule. Efficiency of CD8^+^ T-cell depletion in spleen and Peyer's patch was verified 1 day after two rounds of depleting-antibody injection. For serum transfer, sera collected from VLP-hCEA-treated or control mice were pooled respectively and transferred to 14-week-old *hCEA-Tg/Apc*^*Min/+*^ mice i.p. (200 μl per dose) for 3 times at 2-week intervals. At 2 weeks after the third transfer, mice were killed and intestines were isolated and subjected to intestinal tumour staining as described above.

### Isolation of phagocytes from small intestine

Isolation of small intestinal mononuclear phagocytes was performed as previously described^64^ with modifications. Briefly, small intestine isolated from individual mouse was inverted on a polyethylene tube (BD Biosciences), and washed with calcium- and magnesium-free PBS. Then, the mucus was removed by treating with 1 mM dithiothreitol (Sigma). After wash, the intestine was incubated twice with 30 mM EDTA for 10 min to elute epithelium, followed by incubation with 36 U ml^−1^ collagenase IV (Sigma) and 500 U ml^−1^ of DNase I (Thermo Scientific) in PBS for 90 min at 37 °C. The digested tissue was then passed through a 70-μm Cell Strainer (BD Biosciences) and washed with DMEM. Mononuclear phagocytes were collected from an OptiPrep (Sigma) density centrifugation. Macrophages were enriched by incubating single-cell suspension obtained above with CD11b MACS beads according to the manufacturer's instructions (Miltenyi Biotec). Bead-attached cells were separated by positive selection using MACS LS magnetic column and cultured in RPMI-1640 with 100 U ml^−1^ penicillin, 100 μg ml^−1^ streptomycin and 10% fetal bovine serum.

### CTL assay

The cytotoxicity was measured by a standard 6-h ^51^Cr release assay as previously described^16^,^19^,^65^. Briefly, Peyer's patch lymphocytes were pooled from three to four mice per group and were directly used as effector cells for the mucosal CTL assay. Enriched T cells from individual mouse spleen were *in vitro* stimulated for 5 days and then used as effector cells for the systemic CTL assay. Enriched splenic T cells were cultured in RPMI-1640 with 5 μg ml^−1^ hCEA peptide (CEA526-533 EAQNTTYL) and 5% T-stim without concanavalin A (BD Discovery Labware). Irradiated splenocytes from wild-type C57BL/6 mice were used as feeder cells for *in vitro* stimulation. RMA cells were used as target cells by incubating at 37 °C with 200 μCi sodium chromate (Perkin-Elmer, Boston, MA, USA) for 1 h. Effector cells from Peyer's patches or spleen were seeded into triplicate wells containing the target cells at various effector/target cell ratios in the presence of hCEA peptide or control peptide (LCMV Gp33 KAVYNFATC). Plates were incubated at 37 °C with 5% (v/v) CO_2_ for 6 h. Then, the supernatant was removed from each well and the ^51^Cr release was assessed using a gamma radiation counter (Perkin-Elmer). The calculation of specific cell lysis (%) has been previously described^19^,^65^.

### Enzyme-linked immunosorbent assay

Sera, intestinal washings or faecal extractions from *Apc*^*Min/+*^ or *hCEA-Tg/Apc*^*Min/+*^ mice that were orally immunized with PsV or VLP were used for detecting anti-hCEA antibody response or the presence of mouse albumin by ELISA. For anti-hCEA antibody detection, the plates were coated with 100 μl per well hCEA (Fitzgerald, Concord, MA, USA) at a concentration of 2 μg ml^−1^ overnight. For albumin detection, plates were coated with 1 μg ml^−1^ of anti-albumin capture antibody overnight. Then, wells were blocked with 5% nonfat dry milk in PBS-Tween for 1 h. The plates were then incubated with serially diluted mouse sera, intestinal washings or faecal extractions for 1 h followed by 1 h of incubation with HRP-conjugated anti-mouse IgG (Pierce), HRP-conjugated anti-mouse IgA (Sigma-Aldrich) or HRP-conjugated anti-mouse albumin antibody (Bethyl Laboratories, Inc). 3,3′5,5′-tetramethylbenzidine (Sigma) were used as substrate. The reaction was stopped by 1 M H_2_SO_4_ and the optical density of each well was measured at a wavelength of 450 nm. For the measurement of mouse IL-1β, paired (capture and detection) antibodies and standard recombinant proteins were purchased from eBioscience. Mouse IL-18 was quantified using commercially available ELISA kit (eBioscience) according to the manufacturer's instructions.

### Real-time PCR

Intestinal tissues (nontumour or tumour) were collected from individual mouse in each group for RNA isolation. RNA was isolated and reverse transcribed using Reverse Transcription Supermix (Bio-Rad Laboratories, Inc.). Quantitative real-time PCR analysis was performed in 96-well PCR plates using the CFX96 Touch Real-Time PCR Detection System (Bio-Rad Laboratories, Inc.). All gene expression data are presented as the expression relative to glyceraldehyde-3-phosphate dehydrogenase (GAPDH). The primer sequences for the target genes are obtained from primerbank (http://pga.mgh.harvard.edu/primerbank/) and described in Supplementary Table 1.

### siRNA knocking down

The siRNAs specific for mouse *Aim2* (Santa Cruz Biotechnology, sc-140968 and Life Technologies, 255681) or *Ifi204* (Santa Cruz Biotechnology, sc-40700 and Life Technologies, s68054) were used following the manufacturer's instructions.

### Mitochondrial ROS detection

The measurement of mtROS was as previously described^27^,^66^. Briefly, wild-type BMDMs from *C57Bl/6* mice were treated with VLP for 6 h, and then loaded with 4 μM of MitoSOX (Life Technologies) for 15 min. After that, the cells were washed three times with sterile PBS and subjected to flow cytometric analysis. Mean fluorescence intensity was determined using a FACS Cantoflow cytometer (BD Bioscience), and data were analysed using FlowJo software (Treestar).

### Detection of active caspase-1 *in vivo*

The 14-week-old *Apc*^*Min/+*^ mice were immunized with VLP-hCEA via oral gavage. After 48 h, intestinal tumour polyps from small intestine were collected and snap frozen. Frozen sections were then subjected for co-staining with 4,6-diamidino-2-phenylindole (DAPI), FAM-FLICA caspase-1 probe and A594-conjugated anti-mouse F4/80 (Biolegend) or DAPI, FAM-FLICA caspase-1 probe and anti-mouse EpCAM (Abcam) antibody. A594-conjugated anti-Rat IgG was used as the secondary antibody.

### Detection of PsV infection *in vivo*

VLP-GFP pseudovirus (1 × 10^11^ virus particles) was given to 14-week-old *Apc*^*Min/+*^ mice via oral gavage. After 24 h, tumour polyps from small intestine were isolated and snap frozen. Sections of tissue were subjected for co-staining with DAPI, anti-GFP (Thermo scientific) and A594-conjugated anti-mouse F4/80 (Biolegend) or DAPI, anti-GFP and anti-mouse EpCAM (Abcam) antibody.

### TUNEL staining

The 14-week-old *hCEA-Tg/Apc*^*Min/+*^ mice were orally immunized with VLP-hCEA. At 48 h post immunization, tumour polyps from small intestine were isolated and snap frozen. TUNEL staining was performed in frozen tissue sections to determine the cell death *in vivo*. Co-staining of mouse F4/80 (Biolegend) or mouse EpCAM (Abcam) was performed after TUNEL staining.

### Analysis of caspase-1 activation by western blot

The 14-week-old *Apc*^*Min/+*^ mice were immunized with VLP-hCEA orally. After 24 and 48 h, intestinal tumour polyps were collected and lysed for cellular protein extraction. Protein extractions were then subjected to SDS–polyacrylamide gel electrophoresis, electroblotted to a nitrocellulose membrane and immunoblotted against mouse caspase-1 p20 fragment (AdipoGen) or anti β-actin (Biolegend).

### Analysing the efficacy of IL-18 neutralization *in vivo*

First, to analyse the efficacy of L-18 neutralization *in vivo*, anti-mouse IL-18 neutralizing antibody (R&D system) or a control antibody (rat IgG1) was given to *Apc*^*Min/+*^ mice by i.p. (200 μg per dose) on days −1 and 1 of VLP-hCEA oral immunization. Mice were killed at day 2. In control group, after anti-IL-18 antibody or control IgG1 treatment as described above, LPS (Invivogen) were given to mice i.p. (200 μg per mouse) at day 2 and mice were killed 6 h after injection. Blood and whole small intestines were collected for serum isolation and intestinal homogenization, respectively. Intestines were washed with cold PBS twice and extra liquid were removed by brief spinning. Each small intestine was divided into two parts: upper half and lower half. Intestines were washed with cold PBS twice and extra liquid were removed by brief spinning. Then, intestinal tissues were weighted and homogenized in PBS with 2 × proteinase inhibitor cocktail followed by centrifuge at 5,000 r.p.m. for 10 min. Supernatants were collected and the volume of each sample was measured. IL-18 concentrations in tissue homogenization were measured by ELISA and calculated as pg mg^−1^ tissue. Data are representative of two independent experiments. Data shown as mean±s.d.; *n*=3 in each group. To test the effect of IL-18 in PsV- or VLP-induced tumoridical effect, anti-mouse IL-18 antibody or rat IgG1 isotype control antibody was given to *hCEA-Tg/Apc*^*Min/+*^ mice i.p. (200 μg per dose) on days −1 and 1 of each immunization and every 3 days during the interval of immunization. The mice were under treatment until 2 weeks after final immunization.

### Statistical analysis

All data are shown as mean±s.d. or mean±s.e.m. Statistical significance was analysed by Student's *t*-tests or two-way analysis of variance. Log-rank tests were used for survival analysis. For all tests, *P* values of <0.05 were considered statistically significant.

### Data availability

The authors declare that all the data supporting the findings of this study are available within the article and its supplementary information files and from the corresponding author upon reasonable request.

## Additional information

**How to cite this article:** Zhong, Z. *et al*. Papilloma-pseudovirus eradicates intestinal tumours and triples the lifespan of *Apc*^*Min/+*^ mice. *Nat. Commun.* **8**, 15004 doi: 10.1038/ncomms15004 (2017).

**Publisher's note:** Springer Nature remains neutral with regard to jurisdictional claims in published maps and institutional affiliations.

## Supplementary Material

Supplementary Information Supplementary Figures and Supplementary Table
