# Supplementary material for: Papilloma-pseudovirus eradicates intestinal tumours and triples the lifespan of ApcMin/+ mice
Source: Nat Commun. 2017 Apr 11;8:15004. doi: 10.1038/ncomms15004 (PMC5394268; doi:10.1038/ncomms15004)
Supplement: Supplementary Information — Supplementary Figures and Supplementary Table [file ncomms15004-s1.pdf]

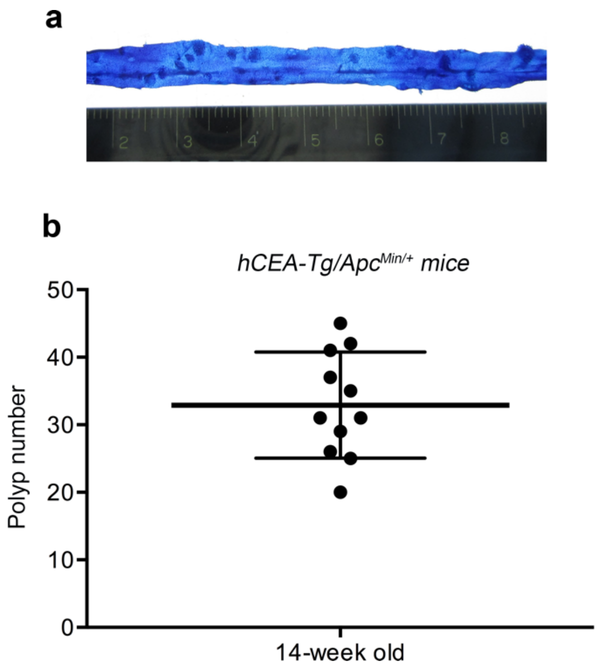

Supplementary Figure 1. Formation of intestinal polyps in 14-week old *hCEA-Tg/Apc*$^{Min/+}$ mice. (**A**) Methylene blue staining of the small intestine from unimmunized *hCEA-Tg/Apc*$^{Min/+}$ mice (14-week old). (**B**) The overall number of intestinal polyps from 14-week old *hCEA-Tg/Apc*$^{Min/+}$ mice (n=11, including 6 male and 5 female mice). Results are shown as mean±s.d.. All data are representative of three independent experiments.

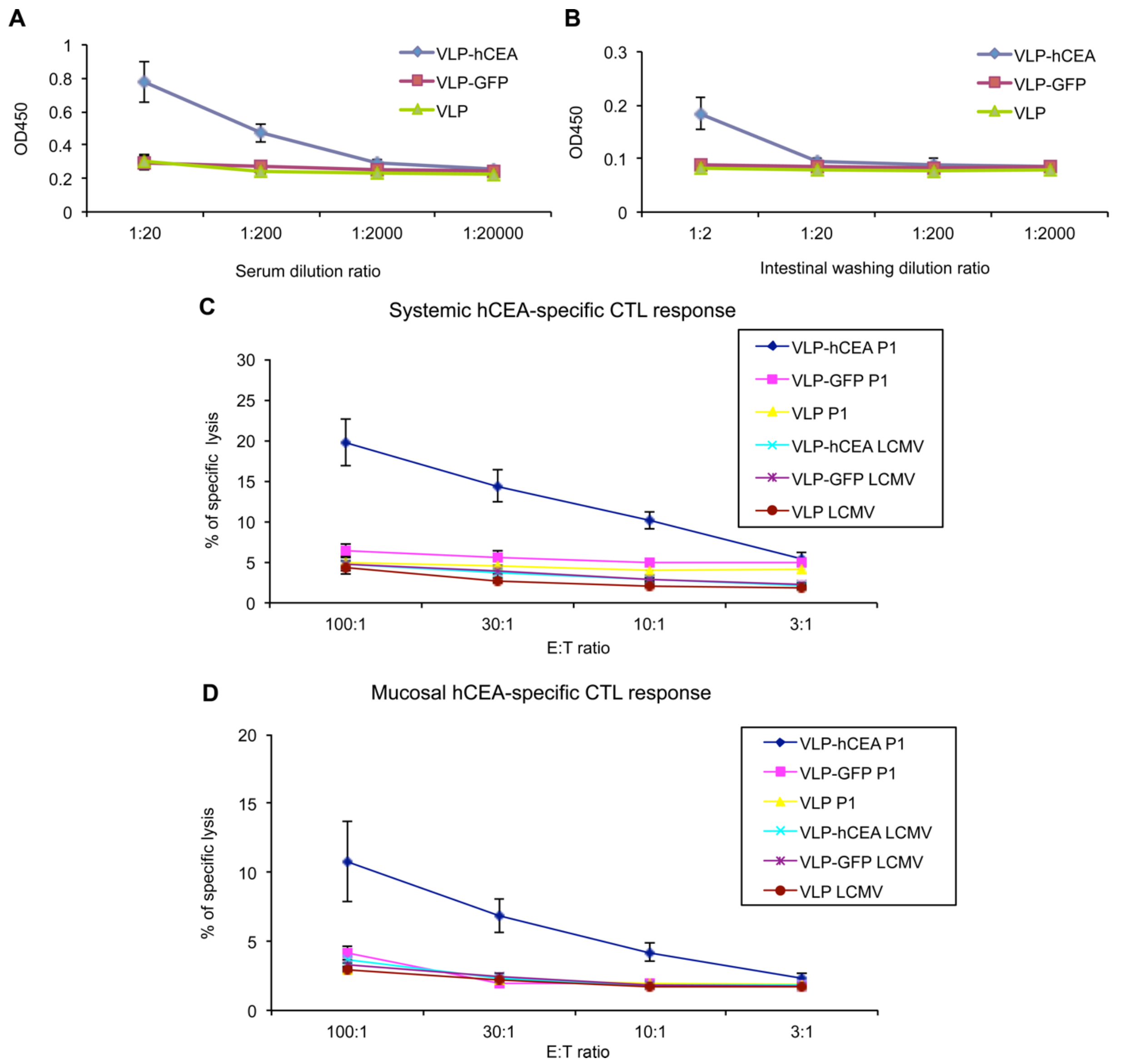

Supplementary Figure 2. VLP-hCEA pseudovirus induces hCEA-specific immunity in *hCEA-Tg/Apc$^{Min/+}$* mice. (**A,B**) The levels of hCEA-specific serum IgG (**A**) and intestinal IgA (**B**) after oral immunization of VLP, VLP-GFP or VLP-hCEA in *hCEA-Tg/Apc$^{Min/+}$* mice. Data in **A** and **B** are shown as mean±s.d. of OD450 values and are representative of three independent experiments. (**C,D**) ^{51}Cr-release assay to determine the splenic or mucosal hCEA-specific CTL responses after VLP, VLP-GFP or VLP-hCEA oral immunization in *hCEA-Tg/Apc$^{Min/+}$* mice. Data in **C** and **D** are shown as mean±s.d. of percentages of specific lysis, and are representative of three independent experiments. P1: hCEA peptide 526-533, EAQNTTYL; LCMV: control peptide Gp33, KAVYNFATC.

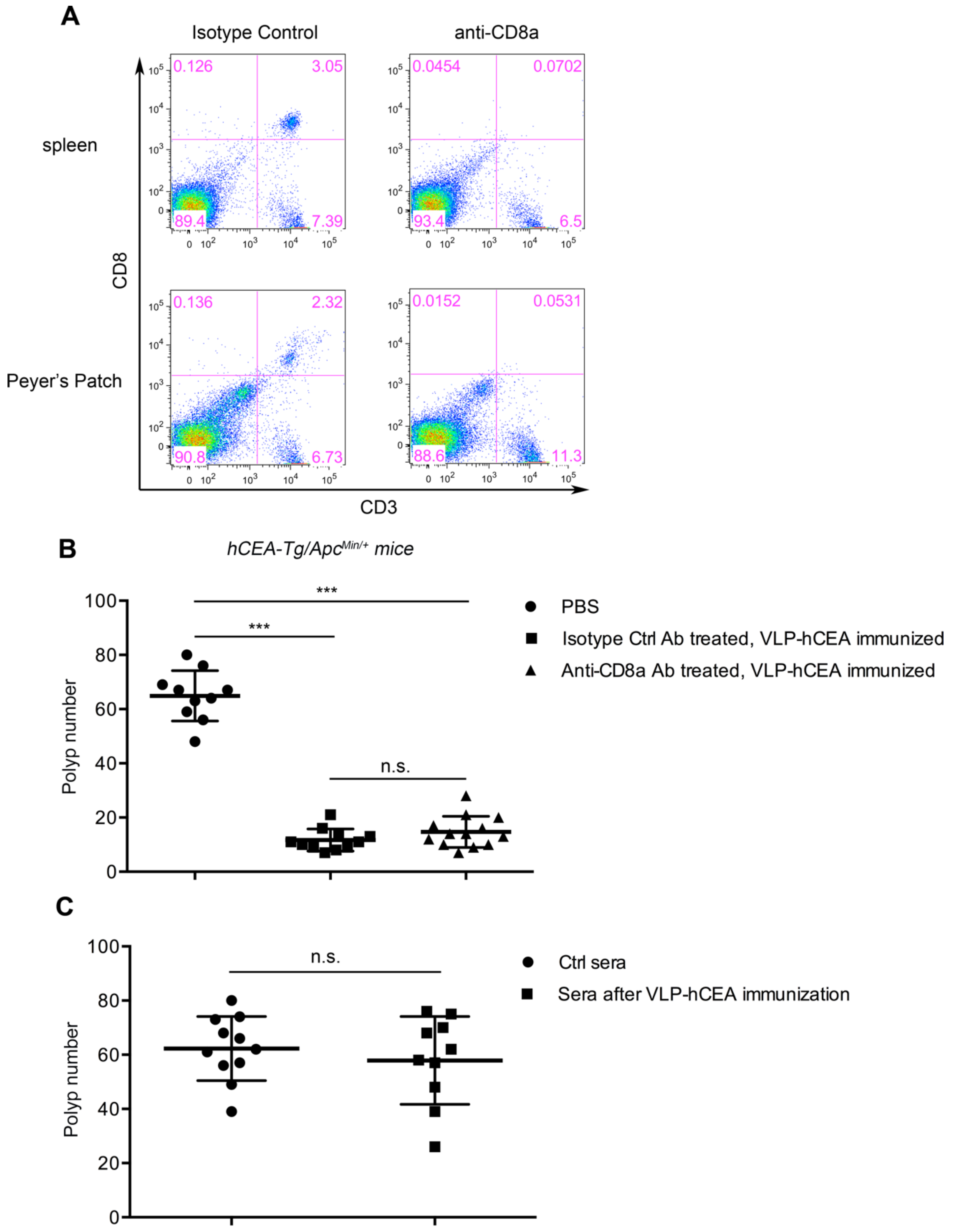

Supplementary Figure 3. CTL response is dispensable for VLP-hCEA-induced tumoricidal effect. (**A**) Representative flow cytometric analysis of $CD8^+$ T cells in spleens and

Peyer's Patches after anti-CD8a neutralizing antibody injection in *hCEA-Tg/Apc*$^{Min/+}$ mice. Briefly, mice were intraperitoneally (i.p.) injected with 250 μg of anti-CD8a IgG2a or isotype control antibodies at 2- and 1-day before every PsV immunization. During the interval of immunization, mice were given additional 250 μg of anti-CD8a antibody every 3 days until the end of the experiment. Efficiency of CD8$^+$ T cell depletion in spleen and Peyer's Patch was verified 1 day after two rounds of antibody injection. Data are representative of three independent experiments. (**B**) The number of intestinal polyps in *hCEA-Tg/Apc*$^{Min/+}$ mice that were pre-deleted of CD8$^+$ T cells followed by VLP-hCEA immunization. Results are shown as mean±s.d.. ANOVA was performed to determine the statistical significance. Data are representative of two independent experiments. (**C**) The intestinal polyp numbers from *hCEA-Tg/Apc*$^{Min/+}$ mice that received either control sera (from untreated *hCEA-Tg/Apc*$^{Min/+}$ mice) or the sera from VLP-hCEA treated (3-dose immunization) hCEA-Tg/Apc$^{Min/+}$ mice. n=10-13, including 4-7 male and 5-6 female mice per group in **B** and **C**. Results are shown as mean±s.d.. Student's t-test was performed to determine the statistical significance. ***, p<0.001; n.s., not significant.

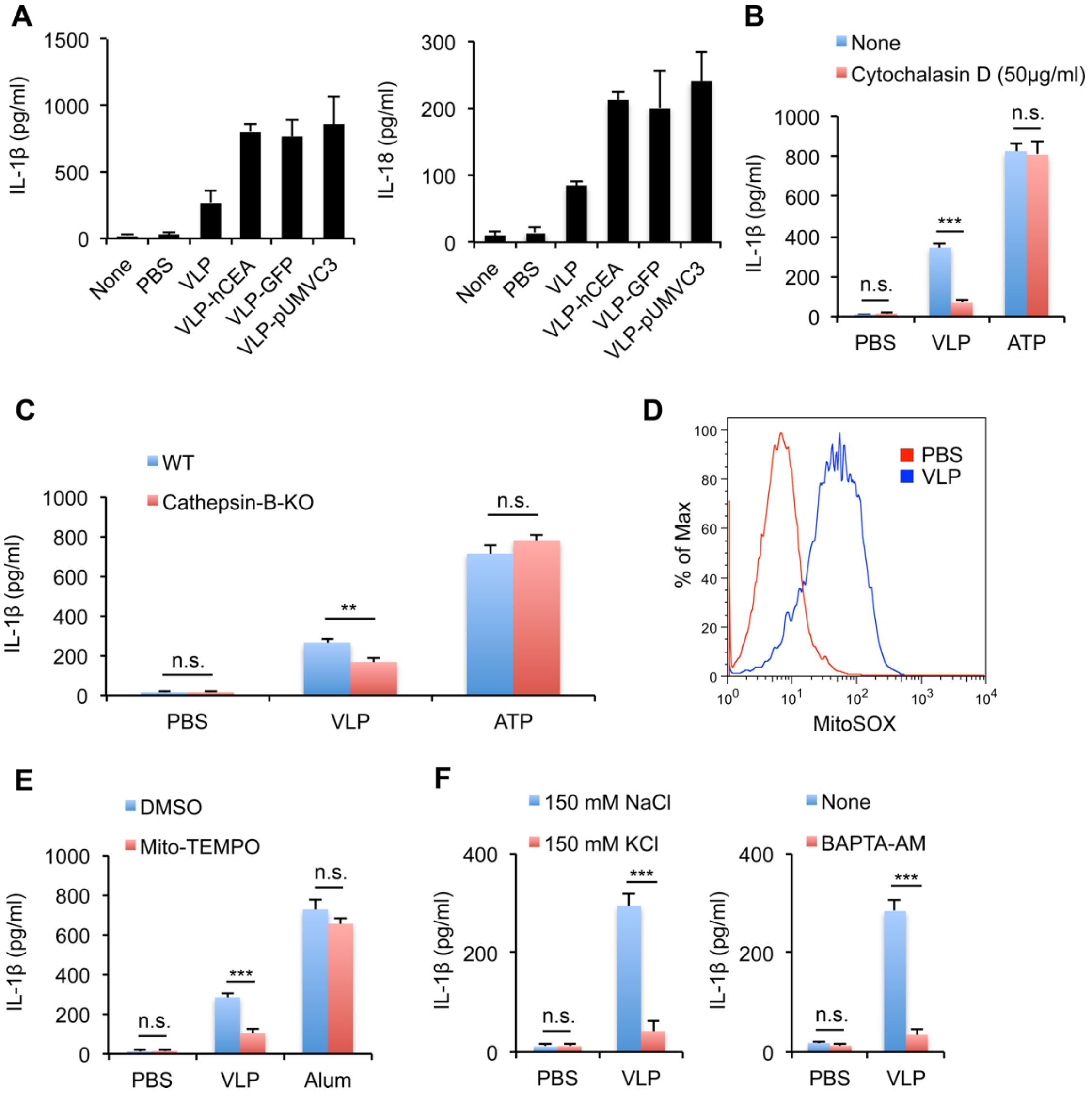

Supplementary Figure 4. VLP activates the NLRP3 inflammasome. (**A**) The levels of secreted IL-1β (left panel) or IL-18 (right panel) from primary BMDMs that were primed with LPS followed by stimulation with stimuli as indicated. Results are shown as mean±s.d., and are representative of three independent experiments. (**B**) IL-1β levels from LPS-primed BMDMs that were pretreated with cytochalasin D followed by VLP or ATP stimulation. Results are shown as mean±s.d.. Student's t-tests were performed to determine the statistical significance. Data are representative of three independent experiments. (**C**) IL-1β levels from LPS-primed immortalized WT or cathepsin-B deficient BMDMs that were stimulated with VLP or ATP. Results are shown as mean±s.d.. Student's t-test was performed to determine the statistical significance. Data are representative of three independent experiments. (**D**) Flow

cytometric analysis of MitoSOX in primary WT BMDMs that were treated with PBS or VLP. Data are representative of three independent experiments. (**E**) IL-1β levels from LPS-primed WT BMDMs that were pretreated with DMSO or Mito-TEMPO followed by VLP or Alum stimulation. Results are shown as mean±s.d.. Student's t-test was performed to determine the statistical significance. Data are representative of three independent experiments. (**F**) The effect of potassium and calcium fluxes in IL-1β secretion in response to VLP stimulation in primary WT BMDMs. Results are shown as mean±s.d.. Student's t-test was performed to determine the statistical significance. Data are representative of three independent experiments. **, $p<0.01$; ***, $p<0.001$; n.s., not significant.

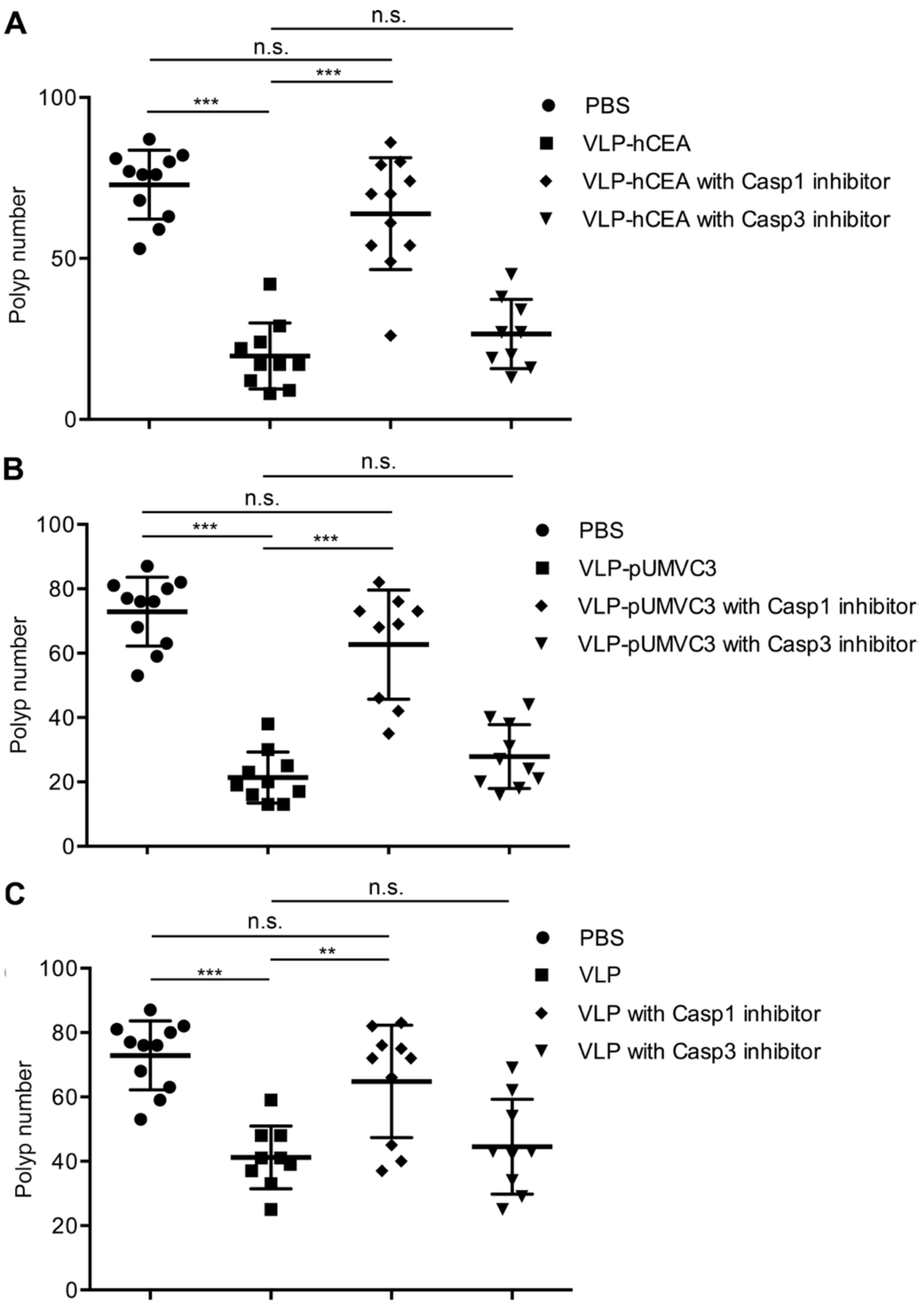

Supplementary Figure 5. Capase-1 mediates the anti-tumor effect of PsV or VLP in *Apc$^{Min/+}$* mice. Numbers of intestinal polyps in *Apc$^{Min/+}$* mice that were treated with caspase-1 or -3 inhibitors followed by immunization with VLP-hCEA (**A**), VLP-pUMVC3 (**B**) or VLP (**C**). n=9-11, including 4-7 male and 4-6 female mice per group. Results are shown as mean±s.d.. ANOVA was performed to determine the statistical significance. Data are representative of two independent experiments. **, p<0.01; ***, p<0.001; n.s., not significant.

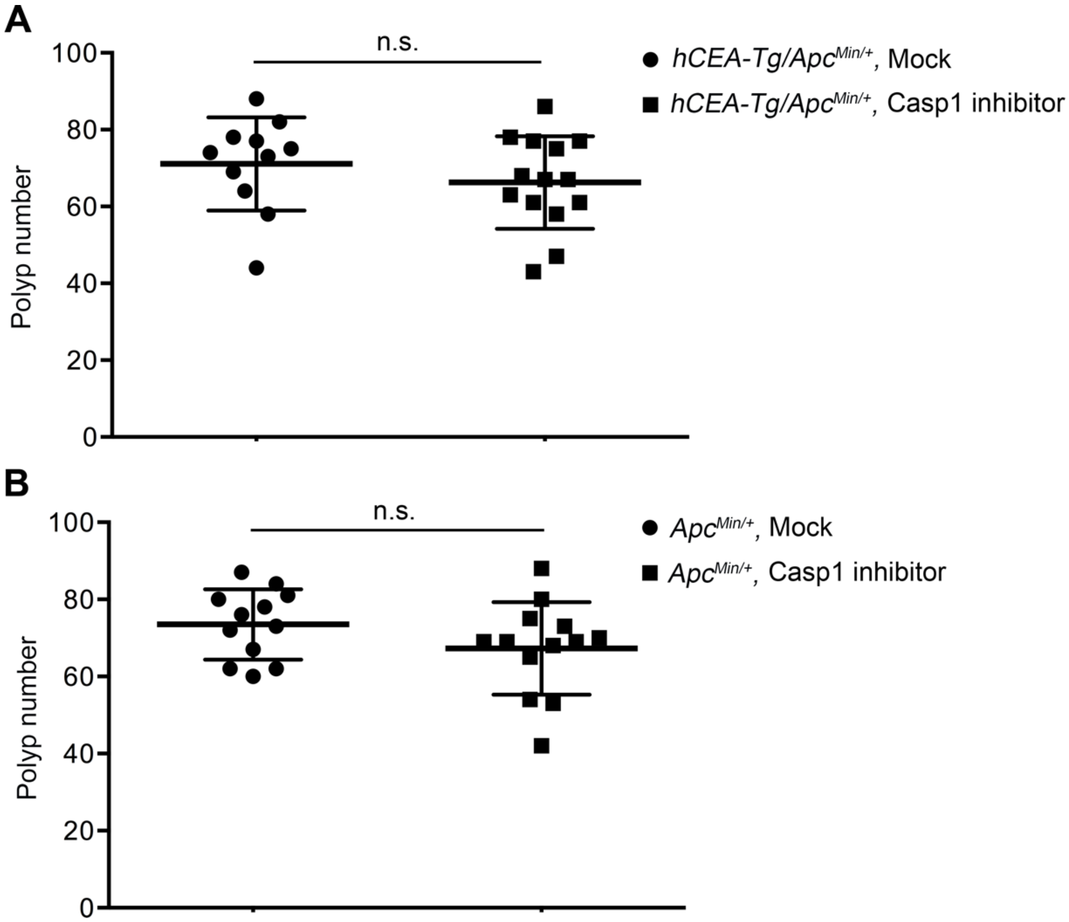

Supplementary Figure 6. Capase-1 does not affect intestinal tumorigenesis in unimmunized *Apc^{Min/+}* mice. Number of intestinal polyps in unimmunized *hCEA-Tg/Apc^{Min/+}* (**A**) or *Apc^{Min/+}* (**B**) mice that were treated with caspase-1 inhibitor. n=11-14, including 6-7 male and 5-8 female mice per group. Results are shown as mean±s.d.. Student's t-test was performed to determine the statistical significance. Data are representative of two independent experiments. n.s., not significant.

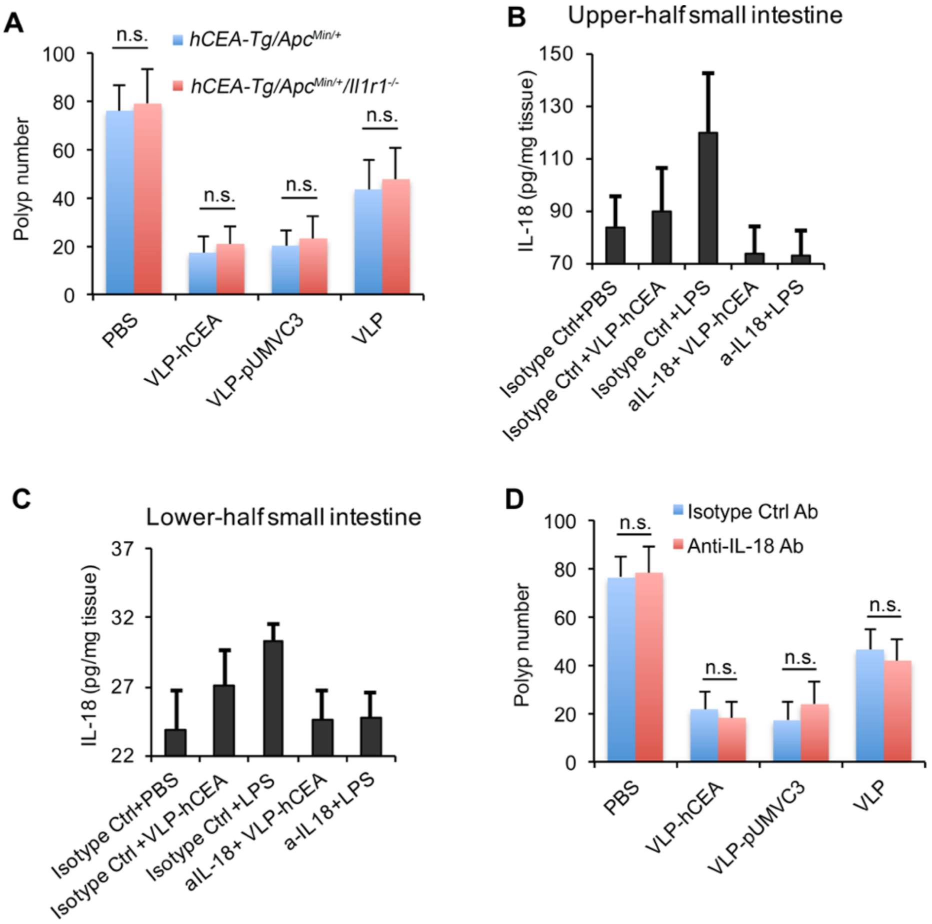

Supplementary Figure 7. IL-1 and IL-18 are dispensable for the PsV-induced anti-tumor effect. (**A**) Numbers of intestinal polys in *hCEA-Tg/Apc*$^{Min/+}$ or *hCEA-Tg/Apc*$^{Min/+}$*/Il-1r1*$^{-/-}$ mice that were orally immunized with PsV or VLP as indicated. n=10-15, including 5-8 male and 5-7 female mice per group. Results are shown as mean±s.d.. Student's t-test was performed to determine the statistical significance. Data are representative of two independent experiments. (**B, C**) Efficacy of IL-18 neutralization. Anti-mouse IL-18 neutralizing antibody or control antibody (rat IgG1) were given to *Apc*$^{Min/+}$ mice by i.p. (200 µg/dose) on days -1 and day 1 of PsV immunization. Mice were sacrificed at day 2. In control group, LPS was given to mice (via i.p. route, 200 µg/mouse) at day 2 and mice were sacrificed 6 hrs after injection. Whole small intestines were collected and divided into 2 parts: upper half (**B**) and lower half (**C**). IL-18 concentrations were measured by ELISA and calculated as pg/mg tissue. Data are representative of two independent experiments and shown as mean±s.d. (n=3, all male mice) in each group. (**D**) Numbers of intestinal polyps of *hCEA-Tg/Apc*$^{Min/+}$ mice that were pretreated with either control or anti-IL-18 neutralizing antibody followed by immunization with PsV or VLP

as indicated. n=9-14, including 5-8 male and 4-6 female mice per group. Results are shown as mean±s.d.. Student's t-test was performed to determine the statistical significance. Data are representative of two independent experiments. n.s., not significant.

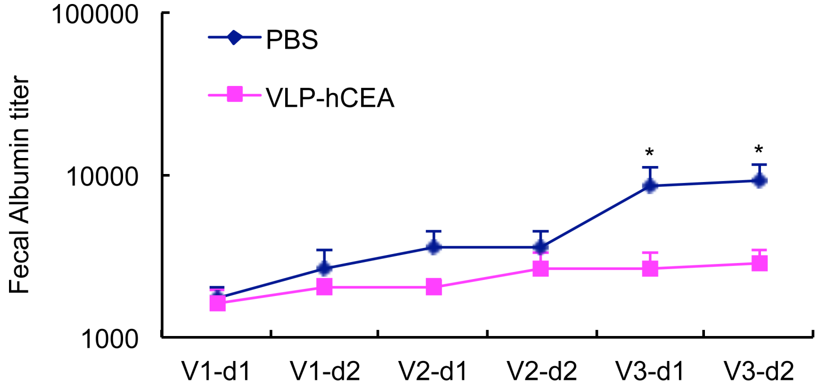

Supplementary Figure 8. PsV do not worsen the gut barrier function in *hCEA-Tg/ Apc$^{Min/+}$* mice. Time lapse [1 or 2 days (d1 or d2) after each treatment (V1, V2, and V3)] measurement of fecal albumin levels from *hCEA-Tg/Apc$^{Min/+}$* mice that were treated with either PBS or VLP-hCEA. n=7 (including 3 male and 4 female mice) for PBS group and n=8 (including 4 male and 4 female mice) for VLP-hCEA group. Results are shown as mean±s.d.. Student's t-test was performed to determine the statistical significance. Data are representative of two independent experiments. *, $p<0.05$.

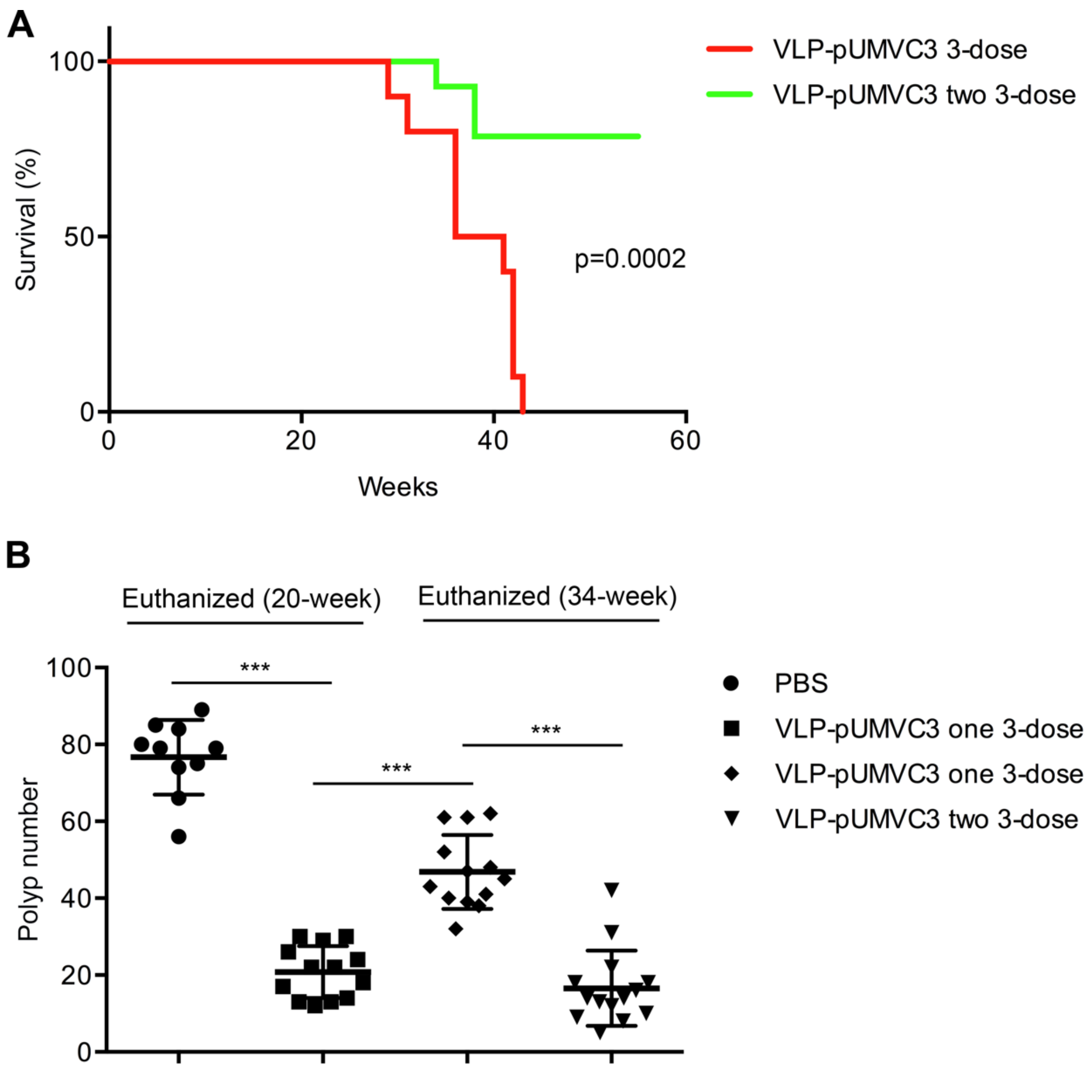

Supplementary Figure 9. PsV immunization frequency positively correlates with the enhanced anti-tumor effect. (**A**) Percentage of survival of *Apc*$^{Min/+}$ mice that were immunized with one (n=10, including 5 male and 5 female mice) or two rounds (n=14, including 6 male and 8 female mice) of 3-dose VLP-pUMVC3 vaccination. Log-rank test was performed to determine the statistic significance. Data are representative of two independent experiments. (**B**) Numbers of intestinal polyps in *Apc*$^{Min/+}$ mice that were immunized with one or two rounds of 3-dose VLP-pUMVC3 vaccination. The mice were euthanized either at 20-week or 34-week of age. n=10-13, including 5-8 male and 5-7 female mice per group. Results are shown as mean±s.d.. ANOVA was performed to determine the statistical significance. ***, p<0.001. Data are representative of two independent experiments.

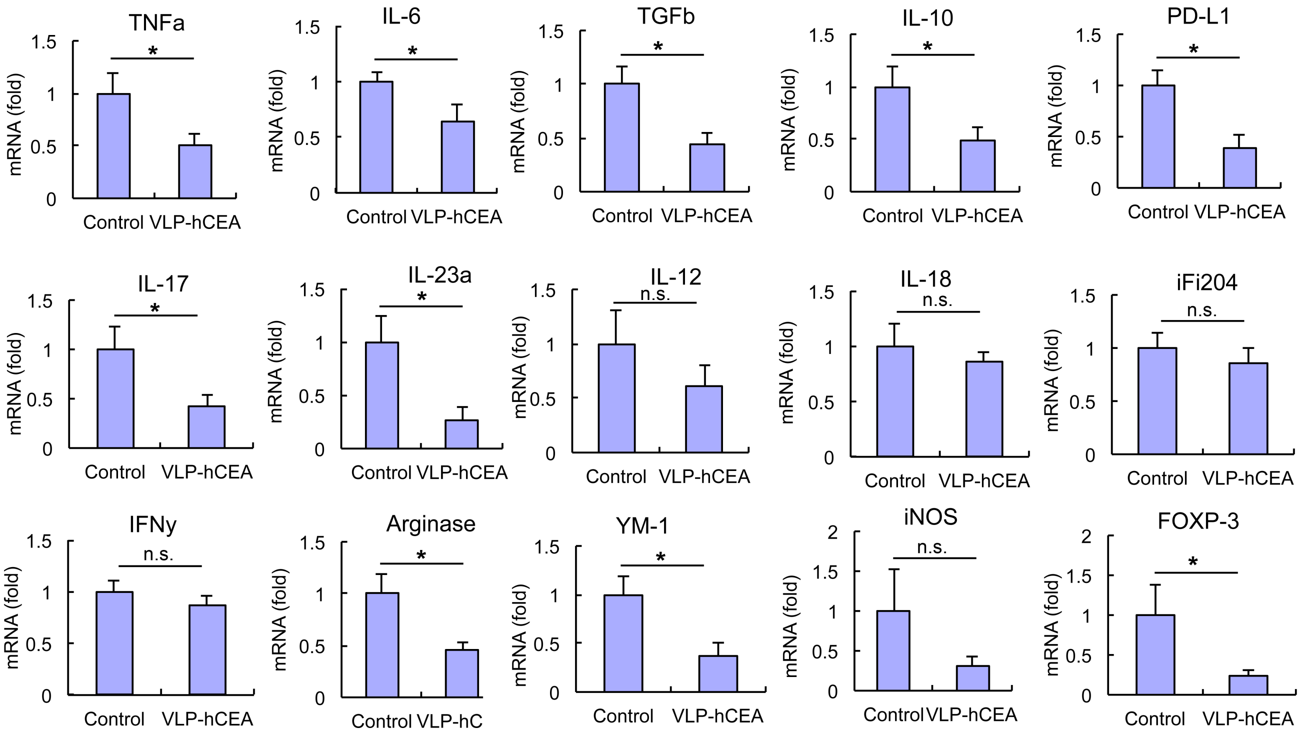

Supplementary Figure 10. VLP-hCEA immunization alters the intestinal tumor microenvironment. 14-week old *hCEA-Tg/Apc*$^{Min/+}$ mice were orally immunized with VLP-hCEA or PBS. 24 hrs post immunization, three small intestinal tumors were collected from individual mice in each group for RNA isolation. Quantitative real-time RT-PCR analysis was performed to determine the expression of indicated genes in tumors. n=3-4 (including 2 female and 1-2 male mice per group). Results are shown as mean±s.d.. Student's t-test was performed to determine the statistical significance. Data are representative of three independent experiments. *, $p<0.05$; n.s., not significant.

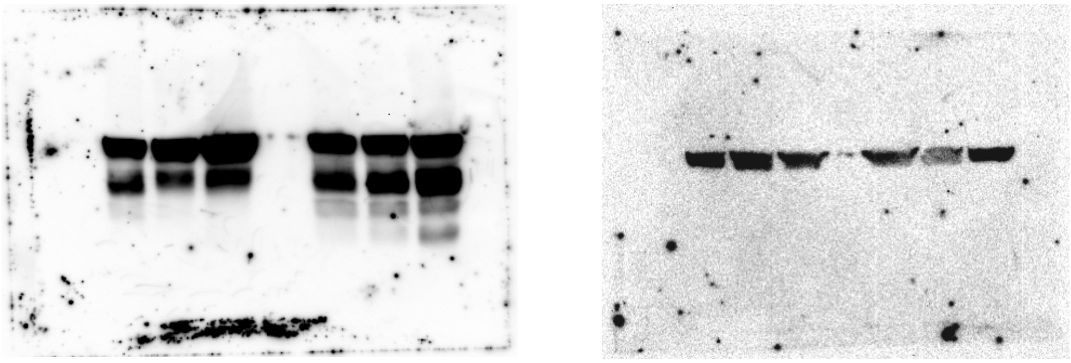

Supplementary Figure 11. Uncropped western blot images of Figure 4D.

Mouse gene	Forward	Reverse
Tnfα	AGGGTCTGGGCCATAGAACT	CCACCACGCTCTTCTGTCTAC
Tgfβ1	CAACCCAGGTCCTTCCTAAA	GGAGAGCCCTGGATACCAAC
Foxp3	CTCGTCTGAAGGCAGAGTCA	TGGCAGAGAGGTATTGAGGG
Ifn-γ	GAGCTCATTGAATGCTTGGC	GCGTCATTGAATCACACCTG
inos	GTTCTCAGCCCAACAATACAAGA	GTGGACGGGTCGATGTCAC
Arginase	TGGCTTGCGAGACGTAGAC	GCTCAGGTGAATCGGCCTTTT
Ym1	TTATCCTGAGTGACCCTTCTAAG	TCATTACCCTGATAGGCATAGG
Il-1β	AGGTCAAAGGTTTGGAAGCA	TGAAGCAGCTATGGCAACTG
Il-18	TCCTTGAAGTTGACGCAAGA	TCCAGCATCAGGACAAAGAA
Il-6	ACCAGAGGAAATTTTCAATAGGC	TGATGCACTTGCAGAAAACA
Il-10	TGTCAAATTCATTCATGGCCT	ATCGATTTCTCCCCTGTGAA
Il-12	GCTTCTCCCACAGGAGGTTT	CTAGACAAGGGCATGCTGGT
Il-17a	TGAGCTTCCCAGATCACAGA	TCCAGAAGGCCCTCAGACTA
Il-23a	GCTCCCCTTTGAAGATGTCA	GACCCACAAGGACTCAAGGA
Pd-L1	GCTCCAAAGGACTTGTACGTG	TGATCTGAAGGGCAGCATTTC
ifi204	TCTTTTCTGGGCTGTGGAAG	ACAGCTCAGGCGAGGACTT
Gapdh	TTGATGGCAACAATCTCCAC	CGTCCCGTAGACAAAATGGT

Supplementary Table 1. Primers sequences for real-time PCR analysis.
